# META-GSA: Combining Findings from Gene-Set Analyses across Several Genome-Wide Association Studies

**DOI:** 10.1371/journal.pone.0140179

**Published:** 2015-10-26

**Authors:** Albert Rosenberger, Stefanie Friedrichs, Christopher I. Amos, Paul Brennan, Gordon Fehringer, Joachim Heinrich, Rayjean J. Hung, Thomas Muley, Martina Müller-Nurasyid, Angela Risch, Heike Bickeböller

**Affiliations:** 1 Department of Genetic Epidemiology, University Medical Center, Georg-August University Göttingen, Göttingen, Germany; 2 Geisel School of Medicine, Dartmouth College, Lebanon, NH, United States of America; 3 International Agency for Research on Cancer (IARC), Lyon, France; 4 Prosserman Centre for Health Research, Samuel Lunenfeld Research Institute, Mount Sinai Hospital, Toronto, Ontario, Canada; 5 Institute of Epidemiology I, Helmholtz Zentrum München, German Research Center for Environmental Health, Neuherberg, Germany; 6 Translational Lung Research Center Heidelberg (TLRC-H), Member of the German Center for Lung Research (DZL), Heidelberg, Germany; 7 Thoraxklinik at University of Heidelberg, Heidelberg, Germany; 8 Department of Medicine I, Ludwig-Maximilians-University Munich, Munich, Germany; 9 Institute of Medical Informatics, Biometry and Epidemiology, Chair of Genetic Epidemiology, Ludwig-Maximilians-University, Munich, Germany; 10 Institute of Genetic Epidemiology, Helmholtz Zentrum München—German Research Center for Environmental Health, Neuherberg, Germany; 11 DZHK (German Centre for Cardiovascular Research), partner site Munich Heart Alliance, Munich, Germany; 12 Division of Epigenomics and Cancer Risk Factors, German Cancer Research Center, Heidelberg, Germany; 13 Division of Molecular Biology, University Salzburg, Salzburg, Austria; Institute for Clinical Epidemiology and Applied Biometry, GERMANY

## Abstract

**Introduction:**

Gene-set analysis (GSA) methods are used as complementary approaches to genome-wide association studies (GWASs). The single marker association estimates of a predefined set of genes are either contrasted with those of all remaining genes or with a null non-associated background. To pool the p-values from several GSAs, it is important to take into account the concordance of the observed *patterns* resulting from single marker association point estimates across any given gene set. Here we propose an enhanced version of Fisher’s inverse χ^2^-method META-GSA, however weighting each study to account for imperfect correlation between *association patterns*.

**Simulation and Power:**

We investigated the performance of META-GSA by simulating GWASs with 500 cases and 500 controls at 100 diallelic markers in 20 different scenarios, simulating different relative risks between 1 and 1.5 in gene sets of 10 genes. Wilcoxon’s rank sum test was applied as GSA for each study. We found that META-GSA has greater power to discover truly associated gene sets than simple pooling of the p-values, by e.g. 59% versus 37%, when the true relative risk for 5 of 10 genes was assume to be 1.5. Under the null hypothesis of no difference in the true *association pattern* between the gene set of interest and the set of remaining genes, the results of both approaches are almost uncorrelated. We recommend not relying on p-values alone when combining the results of independent GSAs.

**Application:**

We applied META-GSA to pool the results of four case-control GWASs of lung cancer risk (Central European Study and Toronto/Lunenfeld-Tanenbaum Research Institute Study; German Lung Cancer Study and MD Anderson Cancer Center Study), which had already been analyzed separately with four different GSA methods (EASE; SLAT, mSUMSTAT and GenGen). This application revealed the pathway GO0015291 “transmembrane transporter activity” as significantly enriched with associated genes (GSA-method: EASE, *p* = 0.0315 corrected for multiple testing). Similar results were found for GO0015464 “acetylcholine receptor activity” but only when not corrected for multiple testing (all GSA-methods applied; p≈0.02).

## Introduction

Genome-wide association studies (GWASs) enable us to identify single markers or narrow genomic regions associated with a disease after genotyping thousands of single nucleotide polymorphisms (SNPs) throughout the whole genome. However, the interplay of genes in the etiology of the phenotype in question has still not been considered. “It is well known that genes do not work in isolation; instead, complex molecular networks and cellular pathways are often involved in disease susceptibility and disease progression” [1]. Hence, to respect pathways, which are typically sets of genes connected through known or proposed mechanisms on a molecular, cellular, or organic level, can help discover genetic susceptibility to complex traits [2].

Gene-set analysis (GSA) was therefore proposed as a complementary approach in the investigation of the genetic basis of disease using GWAS results [3–9]. In principle, GSA either contrasts the entirety of observed marker-specific associations of a common set of genes of interest (*GS*) with those of all remaining genes (*GS´*) (competitive test) or compares these to a null hypothesis (H_0_) of a non-associated background (self-contained test). GSA approaches provide in general no estimate of the strength of association but only p-values (*p*_*GS*_), indicating some kind of accumulation in significance of observed associations with a phenotype for genes or markers within the *GS (*denoted as ***accumulated marker significance***). These *p*_*GS*_-values usually result from one-sided statistical tests. Hence, low *p*_*GS*_–values result from low single marker p-values *p*_*m*_ of genes in *GS*. GSA in general does not provide any estimate of the strength of a joint association for the set of markers or genes in question, respectively. The appeal of GSA as an analysis tool complementary to GWAS is that gene sets which are enriched with moderate association signals may be discovered, even if no individual markers within genes of the *GS* demonstrate genome-wide significance.

If one aims to perform a meta-analysis across several existing GSA studies (*s = 1 …n*_*s*_) based on GWAS results, it seems straightforward simply to apply e.g. Fisher’s inverse χ^2^-method [10] as a quantitative method to pool p-values (*p*_GS,s_) from independent, one-sided tests (further denoted as simple p-pooling (SPP)) of concordant null hypotheses (*direction of the test*) [10]. Nevertheless, low *p*_*GS*_–values can theoretically arise through *accumulated marker significance*, in which the minor alleles of all markers are observed for example as protective factors in one study, while being seen at the same time as risk factors in another study. Thus, significance for *GS* can appear simultaneously in several studies without concordance of the patterns of all observed associations of markers, respectively genes, contained in *GS* (briefly denoted as ***association pattern***). Consequently, concordance of the *direction of the test* (of *p*_*GS*_) is not given *a priori*.

Hence, it is of vital importance to take the *concordance of association patterns* between studies into account when combining *p*_*GS*,*s*_-values of several studies (reflecting *significance of GSs*). Furthermore, replicated *accumulated marker significance* (low *p*_*GS*_–values) alone is insufficient to state the consistency in GSA findings in terms of the Bradford Hill criteria [11].

We propose an approach for a quantitative meta-analysis of GSA results, which we have named META-GSA. This follows the idea of an enhanced version of Fisher’s inverse χ^2^-method [12], weighting each study to account for imperfect correlation between its own *association pattern* with an *overall association pattern* across studies. Because this *overall association pattern* is a hidden construct, we use principal component analysis (PCA) to determine appropriate study weights. Doing so, the weights are neither pre-specified nor strictly positive, as presumed by the above-mentioned method. Thus the distribution of the final test statistic under the H_0_ of “no *accumulated marker significance* taking *concordance of association patterns* into account” needs to be derived by data permutation.

In this manuscript we outline the principle of META-GSA. We then present simulation results considering 20 different scenarios for the “pattern of true marker-phenotype association”, simulating different relative risks between 1 and 1.5 in gene sets of 10 genes. Finally, we apply META-GSA to four lung cancer GWASs from the Transdisciplinary Research in Cancer of the Lung / International Lung Cancer Consortium (TRICL / ILCCO).

## Method

### Notation

**Fig 1:**
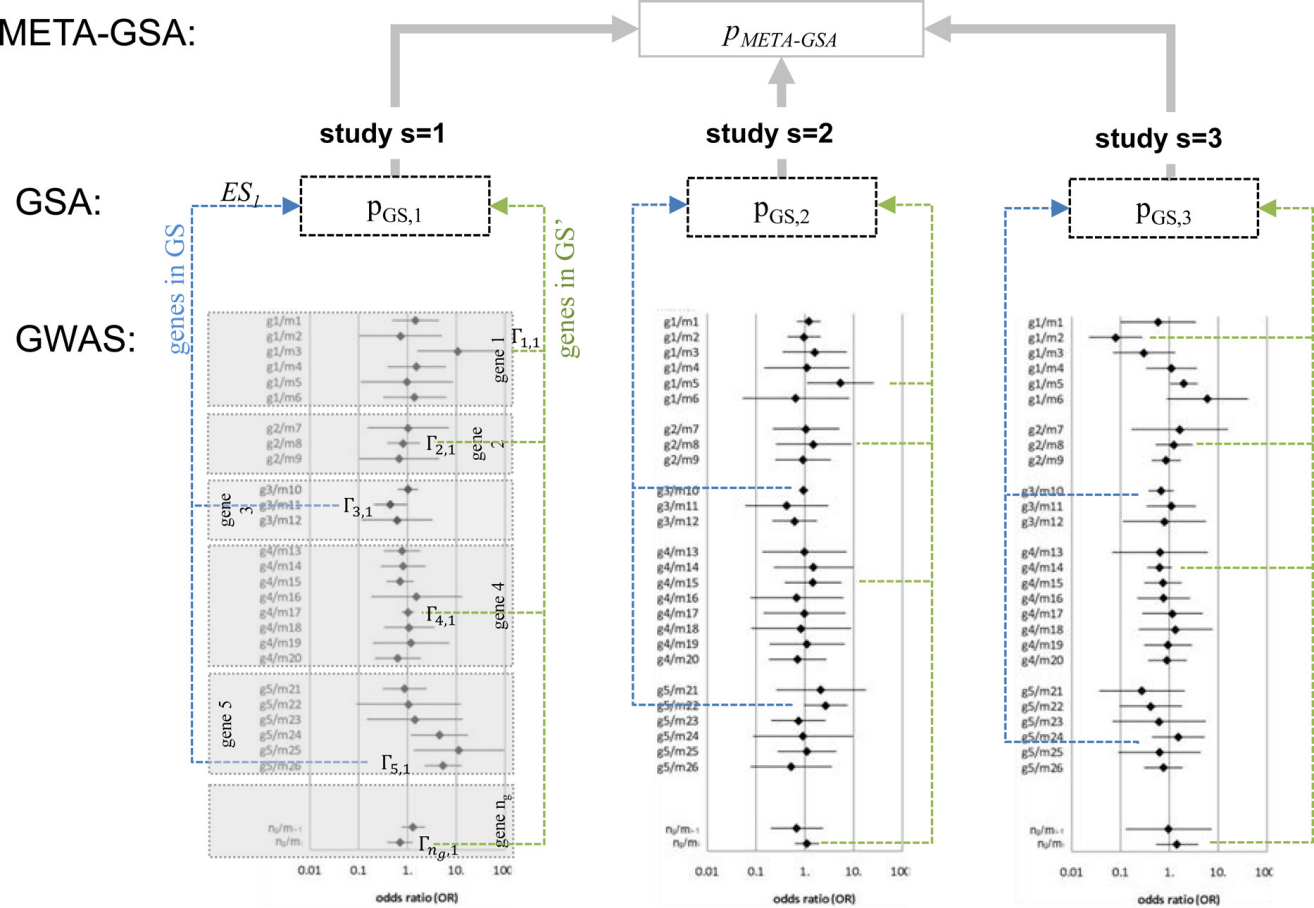
META-GSA: a tool for meta-analysis based on GSAs for GWASs. Genome-wide association study (GWAS); gene-set analysis (GSA); meta-analysis of GSAs (META-GSA); gene set of interest (GS, containing genes 3,5,…); complementary gene set (GS’, containing genes 1, 2, 4,…); gene-level statistic of gene *g* and study *s (Γ*_*g*,*s*_ *)*; “enrichment score” (ES) as test statistic for GSA in study *s*.

Assume that for several independent studies *s = 1…n*_*s*_ gene-set analyses (GSA) based on the results of GWASs have already been performed (see Fig 1). Within each GWAS, a particular association measure *θ*_*m*,*s*_ (e.g. odds ratio OR) for each of *m = 1*,*…*, *n*_*m*_ markers has been estimated. Additionally, corresponding p-values *p*_*m*,*s*_ and/or test statistics *T*_*m*,*s*_ from two-sided tests of no association (in the case of odds ratios the null hypothesis is: H_0_: θ_m,s_ = 1, the alternative hypothesis is H_A_: θ_m,s_≠1) are available. We introduce the parameter *d*_*m*,*s*_ as indicator for the direction of the point estimator of *θ*_*m*,*s*,_ taking values of -1 or +1 (e.g. of *d*_*m*,*s*_ = -1 if *OR*_*m*,*s*_<1 and *d*_*m*,*s*_ = +1 if *OR*_*m*,*s*_≥1).

Within each GSA, a common set of genes *GS*, identical for all studies s, was compared to another set *GS`*of genes, possibly all remaining genes or to a non-associated background. A gene-level statistic Γ_g,s_ for each gene *g* in each study s was constructed from *θ*_*m|g*,*s*_, *T*_*m|g*,*s*_ or *p*_*m|g*,*s*_, respectively (e.g. the lowest observed marker-specific p_m|g,s_-value for all markers of gene *m|g*). A GSA test statistic for each study *ES*_*s*_, e.g. an enrichment score, was calculated from Γ_g,s_. Usually, p-values *p*_*GS*,*s*_ *(ES*_*s*_ *)* are gained from one-sided tests of H_0,s_: ES_s_ = 0 versus e.g. H_A,s_: ES_s_>0, i.e. the alternative is an enrichment of low *p*_*m|g*,*s*_-values of genes from *GS*.

A meta-analytical approach with several levels such as markers, genes, and gene sets is necessarily rich in notation. Thus the relationship between GWAS, GSA, and META-GSA is summarized in Fig 1. All the important notation is listed in Table 1.

**Table 1:** Notation.

*n*_*s*_	no. of studies		
*s = 1 to n*_*s*_	study		
*n*_*m*_	no. of markers		
*m = 1 to n*_*m*_	marker		
*r²*_*m*,*m´*_	measure of linkage disequilibrium	between marker *m*	and *m´*
*n*_*g*_	no. of genes		
*g = 1 to n*_*g*_	gene		
*n*_*m|g*_	no. of markers assigned to gene g		
*θ*_*m*,*s*_	association measure (odds ratio)	for marker *m*	of study *s*
*d*_*m*,*s*_	direction of *θ*_*m*,*s*_ ∈ [−1,+1]	for marker *m*	of study *s*
*p*_*m*,*s*_	p-value of testing e.g. H_0_: θ_m,s_ = 1	for marker *m*	
*p*_*g*,*s*_	p-value of a gene	for gene g	of study *s*
*d*_*g*,*s*_	direction of a gene	for gene g	of study *s*
*p´*_*g*,*s*_	directed reverse p-value (PDR)	for gene *g*	of study *s*
*n*_*GS*_	no. of gene sets		
*GS = 1 to n*_*GS*_	gene set of interest		
*GS`*	gene set complimentary to *GS*		
*Γ*_*g*,*s*_	Gene-level statistic of GSA	for gene *g*	of study *s*
*ES*_*GS*,*s*_	test statistic of GSA	for gene set *GS*	of study *s*
*p*_*GS*,*s*_	p-value of GSA	for gene set *GS*	of study *s*
$M_{n_{s}}$	test statistic of META-GSA	for gene set *GS*	across studies
*p*_*permut*,*GS*_	p-value of META-GSA (nominal)	for gene set *GS*	across studies
*p*_*META-GSA*,*GS*_	p-value of META-GSA (adjusted for multiple testing)	for gene set *GS*	across studies
There are four tests mentioned further:			
*SPP*	testing significance of simple p-pooling	unconditional combination of *p*_*GS*,*s*_	
*direction test*	testing concordance of association patterns		
pooledGWAS-GSA		first performing a random effects meta-analysis for each marker and then perform a single GSA	
META-GSA		conditional combination of SPP and the direction test	

The term *nominal significance* is used to indicate significance without correcting for multiple testing.

### Aim and principle of META-GSA

The **aim** of META-GSA is to increase statistical evidence by pooling *p-values* of GSAs (*p*_*GS*,*s*_ reflecting ***GS-significance*,** this is equivalent to *accumulated marker significance* **)**, taking also into account the ***concordance of association patterns*** (reflected by the *magnitude* of *p*_*m|g*,*s*_ and the *direction* of *θ*_*m|g*,*s*_). We only consider situations in which all studies use the same association measure (e.g. odds ratio).

A well-known *p-pooling* technique to combine evidence from independent one-sided tests of significance is *Fisher’s inverse χ* ^2^ *-method* or enhancements thereof [10, 12, 13]. Assuming a p-value *p* to follow a uniform distribution, if H_0_ is true, the sum $M_{n_{s}}=-2\sum_{s=1}^{n_{s}}\ln(p_{s})$ of *n*_*s*_ independent tests then follows a χ^2^-distribution, with *df = 2n*_*s*_ degrees of freedom. $M_{n_{s}}$ is meaningful if and only if all related test statistics *T*_*s*_ point towards the same direction in terms of the target measure. Makambi enhanced this method by introducing pre-specified weights *w*_*s*_ for each study ($\sum_{s=1}^{n_{s}}w_{s}=1$ and *w*_*s*_ > 0 for all *s*), to account for imperfect concordance of the *direction of the test* [12]. More details are given in S1 and S2 Figs and S1 Text.

The **principle** of META-GSA is to make use of the test statistic $M_{n_{s}}=-2\sum_{s=1}^{n_{s}}w_{s}\ln(p_{GS,s})$ by allocating both necessary aspects (*GS-significance* and *concordance of association patterns*) to its parameters:

○ *p*_*GS*,*s*_ representing *GS-significance*:
Testing in GSA is usually performed one-sided, thus study-specific *p*_*GS*,*s*_ are used as the p-values to be combined.
○ *w*_*s*_ representing *concordance of association patterns*
(patterns of all or a selected number of observed marker-specific associations of markers, respectively genes, contained in *GS*)

### PDR: A single quantity combing significance and direction of association

A core element of META-GSA is the combination of significance (p-values: *p*_*g*,*s*_) and direction (e.g. sign of association measure) of an observed gene-specific association in a manner similar to a correlation coefficient, such that zero represents no evidence and the limits +1 and -1 represent strong evidence of positive or negative association. Thus we define a *directed reversed p-value* (PDR)

as

$$p_{g,s}^{\prime }=d_{g,s}\cdot (1-p_{g,s})$$ (1)

where

$$d_{m,s}=direction\;of\;g\in \left\{\text{-}1,+1\right\}$$ (2)

This definition proved superior to two alternative definitions, which are presented in S2 Text.

### The workflow of META-GSA

To perform META-GSA, the following four steps are necessary to select and concentrate marker-specific information up to study-specific weights *w*_s,_ to determine the null distribution of the test statistics $M_{n_{s}}$, as well as to assess the overall significance:

step I) From the marker- to the gene-level:
Combination of significance (p-value) and direction (sign of effect estimate) to a directed reverse p-value (PDR) for each gene;
step II) From the gene-level to between-study concordance:
Determination of study weights from PDRs,
step III) Performance of significance testing for a GS and
step IV) Correction for multiple testing.

Although summarizing marker-specific association results is necessary as an intermediate step when performing META-GSA, it is not our aim to perform gene-level tests of association. Gene-level statistics are considered only to assess the *concordance of association patterns* between studies.

### Step I: Combination of significance and direction to a PDR for each gene

Marker information is aggregate to a gene-level statistic for each gene and each study by calculating PDRs $p_{g,s}^{\prime }$, since often several markers are allocated to a gene. In general, there are three possible strategies of marker selection / aggregation with the aim of calculating PDRs:

○ Choosing the best (most significant) marker for a gene
○ “Averaging” over all markers allocated to a gene
○ “Averaging” over only “promising” markers allocated to a gene

For META-GSA it is advisable, but not essential, to apply the same strategy of marker selection / aggregation that was used for individual GSAs.

This step, as stated previously, is solely to assess the *concordance of association patterns*. This is the purpose of *“PDR-profiles”*, one for each study, comprising all genes in GS.

#### Choosing the best (most significant) marker for a gene

To choose the most significant marker ${\ddot{m}}_{s}$ per gene as *representative marker* is the easiest way to “aggregate” multiple marker information to a single gene-level statistic within each study [8, 14].

Gene-level significance *p*_*g*,*s*_: In such a case, the p-values for a gene (within each study) is give straightforward:

$$p_{g,s}=\min(p_{m|g,s})$$ (3a)

Gene-level direction d_g,s:_ The determination of the direction *d*_*g*,*s*_ is a bit more complicate. In the simplest case, all *representative markers* ${\ddot{m}}_{s}$ *are the same* across the studies. The direction *d*_*g*,*s*_ is then the sign of the association point estimate $\theta_{\ddot{m},s}$ (if *H*_0_: *θ*_*m*,*s*_ = 0 as for log(odd ratio)):

$$d_{g,s}=sign\left(\theta_{\overset{¨}{m},s}\right).$$ (3b)

If the *representative markers* ${\ddot{m}}_{s}$ *are not the same*, LD between these markers needs to be taken into account to determine *d*_*g*,*s*_. As measure of choice for LD, we consider *r* the correlation between the alleles of marker ${\ddot{m}}_{s}$ and ${\ddot{m´}}_{s´}$. A motivation for this choice is given in S3 Text.

To compute the direction *d*_*g*,*s*_ for study *s*, we multiply then sign of $\theta_{\ddot{m},s}$ with the mean of all *r* of the respective marker ${\ddot{m}}_{s}$ with those markers selected by any other study ${\ddot{m}´}_{s´}$:

$$d_{g,s}{=sign(\theta }_{\overset{¨}{m},s})∙\bar{r_{\overset{¨}{m}|s,\overset{¨}{m}´|s´}}$$ (3c)

Please note, if the representative markers of one study ${\ddot{m}}_{s}$ is in linkage equilibrium to any selected marker for another studies ${\ddot{m}´}_{s´}$, the direction *d*_*g*,*s*_ = *0*. Regardless of the observed significance $p_{\ddot{m},s}$ of this marker the PDR will get $p_{g,s}^{\prime }=0$.

#### “Averaging” over all markers allocated to a gene

Another way to generate a single gene-level statistic is to combine evidence for association of all markers assigned to a gene [14].

*Gene-level significance p*_*g*,*s*_: Performing the weighted Fisher inverse χ^2^-method for single marker p-values *p*_*m|g*,*s*_ is one possibility. Thus the sum $C_{g,s}=\text{-}2\sum_{i=1}^{n_{m|g,s}}v_{i}ln(p_{i,s})$ is calculated and a p-value *p*_*g*,*s*_ is derived from a χ^2^-distribution. Weights *v*_*m|g*,*s*_ are assigned to markers in order to account for pairwise LD and the LD-block structure, respectively. For mSUMSTAT [15] or SLAT [16] permutation approaches to determine these marker weights were previously proposed, but these are time consuming. Instead we choose *v*_*m|g*,*s*_ proportional to the inverse of the sum of *r* ^2^ across any pair of markers in the same LD-block: $v_{m|g}=1/\sum_{m´}r_{m´|g,m|g}^{2}$ (m´ indicating any marker within the same LD-block). Thus the sum of marker weights ∑_*g*,*s*_ *v*_*m*|*g*,*s*_ is equal to the “number of independent markers” which also defines the degrees of freedom for the considered χ^2^-distribution.

Hence, the p-values for a gene (within each study) is, in contrast to Eq 3a, a function of all markers:

$$p_{g,s}=f_{m|g}(p_{m|g,s})$$ (4a)

*Gene-level direction d*_*g*,*s*_: The direction *d*_*g*,*s*_ is simply determined as the weighted mean of marker specific direction (as calculated according Eq 3c), using the same marker weights *v*_*m|g*,*s*_ as before:

$$d_{g,s}=∙\sum_{i=1}^{n_{m|g}}v_{m|g,s}{sign(\theta }_{i,s})$$ (4b)

#### “Averaging” over only “promising” markers allocated to a gene

The same procedures as just explained can be applied restricted to “promising” markers (e.g. filtered for *p*_*m*|*g*,*s*_<0.05).

#### Re-orientate the gene-level directions *d*_*g*,*s*_ across studies

To be able to calculated comparable PDRs, one needs to re-orientate these gene-level directions *d*_*g*,*s*_ along a “profile of reference directions”, represented by the mean of directions across studies for each gene: $\bar{d_{g,*}}=\frac{1}{n_{s}}\sum_{s=1}^{n_{s}}d_{g,s}$. In the case the $\bar{d_{g,*}}$ is negative for a gene g*, all *d*_g*,*s*_ (direction within a study) are multiplied with -1, to ensure a positive value indicating, that a marker is pointing towards the reference direction (mean of all studies).

As soon as the direction *d*_*g*,*s*_ and the significance *p*_*g*,*s*_ of all genes and for each study are determined, PDR-values $p_{g,s}^{\prime }$ can be calculated according to Eq 1 and comprised (for genes in GS only) in a vector of observed gene-phenotype associations $p_{GS,s}^{\prime }=\left(p_{g|GS,s}^{\prime }\right)$. It is important to note that a true “*PDR-profile”* of the *GS* of interest is unknown. The average profile is used as reference instead.

### Step II: Determination study weights *w*_*s*_ from rank correlations of PDRs

#### Determination of the rank correlation of PDRs between studies

Next, we have to quantify the pairwise concordance of observed *PDR-profiles* between studies. For this, we simply calculate all pairwise correlations *τ*_*s*,*s’*_.

Regarding $p_{g,s}^{\prime }$ as a function of the true association *θ*_*m*,*s*_, the sample size *n*_*m*,*s*_, perhaps population stratification, and the fitted statistical model, PDRs may be “scaled” differently between studies. Hence, we propose applying Kendall’s τ. Alternative correlation measures were also investigated (see S2 Text).

#### Deriving study weights *w*_*s*_ by principal component analysis

Once the correlation matrix is found, we use principal component analysis (PCA) to determine the load of each study on a common but unknown general *PDR-profile* of the *GS*. We assume that these loads can be represented by the first principal component (*PC1*_*s*_). The corresponding eigenvalue (*EV1*) is *n*_*s*_-times the fraction of the variance accounted for by *PC1*_*s*_. Only in the case of perfect pairwise correlation between (total *concordance* of) PDR-profiles $p_{GS,s}^{\prime }$ between all studies *s*, this proportion will be 1 and hence *EV1* = *n*_*2*_ and *PC1*_*s*_ = *1*. We interpret the fraction $\frac{EV1}{n_{s}}$ as “*effective number of studies”*.

Finally, a study weight *w*_*s*_ is calculated as the product of the *normalized load* and the *effective number of studies* (= fraction of explained variance):

$$w_{s}=\frac{{PC1}_{s}}{\sum_{j=1}^{n_{s}}{PC1}_{j}}∙\frac{EV1}{n_{s}}$$ (5)

This satisfies the condition that the sum of weights is equal to the *effective number of studies:* $\sum_{s=1}^{n_{s}}w_{s}=\frac{EV1}{n_{s}}$. However this does not satisfy $\sum_{s=1}^{n_{s}}w_{s}=1$ and *1*≥*w*_*s*_≥*0* for all *s* as would be necessary for Makambi’s enhancement of Fisher’s inverse χ^2^-method.

### Step III: Performing significance testing

The assumption that the test statistic $M_{n_{s}}$ asymptotically follows a *χ* ^2^-distribution is based on a few conditions: The p-values are independent, identically distributed (iid), and follow a uniform distribution under H_0_. The weights *w*_*s*_ are pre-specified, range between 0 and 1, and sum up to one ($\sum_{s=1}^{n_{s}}w_{s}=1$ and *w*_*s*_≥*0* for all s). Thus the test statistic $M_{n_{s}}$ is strictly non-negative: $M_{n_{s}}\geq 0$.

Since the assumptions for *w*_*s*_ and $M_{n_{s}}$ are not fulfilled, the null distribution of the test statistic *M*_*0*_ needs to be determined by a permutation procedure, permuting the allocation of genes to *GS* (to determine *w*_*s*_) and drawing *p*_*GS*,*s*_-values by random from a uniform distribution. The statistical significance is expressed by the permutation p-value *p*_*permute*,*GS*_, which is assessed by the fraction of permutations achieving a test statistic *M*_*j*_ (j = 1 to x) at least as extreme as the non-permuted (original) test statistic M_0_. Details and techniques to reduce the computational burden are given in S4 Text and S5 Text.

### Step IV: Correction of multiple testing

Correction for multiple testing is required if several GSs have been investigated. Because a single genes can appear across multiple gene sets and genes can be in LD to each other, *p*_*permut*,*GS*_-values are maybe not independent. Application of a Bonferroni or Šidák adjustment for multiple testing would lead to overcorrection. We propose applying the bootstrap method of Storey and Tibshirani [17, 18] to estimate the proportion of true null hypotheses *π*_*0*_ and use this in a Bonferroni-like manner to correct p-values for each *GS*:

$$p_{META\text{-}GSA,GS}{=min(1,p}_{permute,GS}∙n_{GS}∙\pi_{0})$$ (6)

Details of how *π*_0_ is derived are given in S6 Text.

## Simulation

### Method

We performed power simulations in order to compare the performance of META-GSA and the simple p-pooling of GSA results. To this end, we set up 20 different scenarios for “patterns of true marker-phenotype associations” investigated by GSAs based on GWAS data in two to ten studies (see Table 2). For simplicity, we assumed only one genotyped marker for each gene. These patterns comprised the following situations (H_0_ and H_A_ are expressed according to a competitive test strategy):

1. H_0_: no enrichment in the *GS* of interest: F(RR|GS) = F(RR|GS´) scenarios 1–3
2. H_A_: markers with RR>1 only in *GS*: F(RR|GS)≠F(RR|GS´) scenarios 4–16
3. H_A_/H_0_: mixed structured gene sets (markers with RR≠1 in *GS* and *GS’*) scenarios 17–20

where F(RR|GS) is the cumulative distribution function of “true” relative risks (RR) of markers/genes in *GS*. For example, in scenario 1 the true relative risk (RR) of all markers was set to RR = 1, in scenario 2 to RR = 1.2. Both scenarios represent a H_0_ situation for a competitive test strategy. In scenario 5 we assumed RR = 1.2 for half of the markers of *GS* and a RR = 1 for the other half and all of the markers of *GS´*. Not necessarily the same markers of *GS* needed to be truly associated in each study. However, a true association only appears in genes belonging to the gene set of interest, which is an idealized situation for the competitive test strategy.

**Table 2:** Power of META-GSA, pooledGWAS-GSA and SPP across all studies, based on 100 genes with 1 marker each.

patterns of true marker-phenotype associations							
*scenario no*.		*RRs in GS of interest*	*RRs in complementary GS´*	*no*. *studies*	SPP	pooledGWAS-GSA	META-GSA
**H**_**0**_ **: F(RR|GS) = F(RR|GS´)**							
**1**	no gene is associated at all	10x1	90x1	10	5.6%	3.7%	4.4%
**2**	all genes are associated	10x1.2	90x1.2	10	5.2%	5.5%	7.0%
**3**	all genes are associated	10x1.5	90x1.5	10	6.0%	5.6%	4.8%
**H**_**A**_ **: RR>1 only in *GS* / F(RR|GS)≠F(RR|GS´)**							
**4**	½ the genes in *GS* are associated	5x1 5x1.1	90x1	10	5.4%	6.7%	9.2%
**5**		5x1 5x1.2	90x1	10	9.4%	9.5%	26.4%
**6**		5x1 5x1.3	90x1	10	21.0%	20.7%	48.8%
**7**		5x1 5x1.4	90x1	10	29.6%	31.3%	58.0%
**8**		5x1 5x1.5	90x1	10	36.8%	56.4%	58.8%
**H**_**A**_ **: RR>1 only in *GS* / F(RR|S)≠F(RR|NS)–increasing number of studies**							
**9**		5x1 5x1.5	90x1	2	16.0%	12.7%	24.4%
**10**		5x1 5x1.5	90x1	3	16.8%	15.9%	31.2%
**11**		5x1 5x1.5	90x1	4	27.2%	21.3%	41.8%
**12**		5x1 5x1.5	90x1	5	27.8%	25.0%	45.0%
**13**		5x1 5x1.5	90x1	6	28.4%	30.2%	47.4%
**14**		5x1 5x1.5	90x1	7	31.8%	38.2%	50.4%
**15**		5x1 5x1.5	90x1	8	34.4%	40.9%	54.4%
**16**		5x1 5x1.5	90x1	9	35.6%	47.2%	55.2%
**H**_**A**_ **/H**_**0**_ **: mixed structured *GS***							
**17**	H_A_: assoc. genes in *GS* only	5x1 4x1.2 1x1.5	90x1	10	17.0%	16.1%	35.6%
**18**	H_A_: GS dominates *GS’*	5x1 4x1.2 1x1.5	63x1 18x1.2 9x1.5	10	11.0%	11.4%	5.8%
**19**	H_A_: GS is dominated^**§**^ by *GS‘*	5x1 4x1.2 1x1.5	41x1 32x1.2 17x1.5	10	8.4%	1.7%	1.6%
**20**	H_0:_ same prop. of genes are associated in *GS* and *GS’*	5x1 4x1.2 1x1.5	45x1 36x1.2 9x1.5	10	4.4%	4.7%	3.0%

Given a true type I error of 5%, the observed type I error may range from 3% to 7% (95% random dispersion interval for 500 simulations). Given a true power of 50%, the observed power may range from 45% to 54% (95% random dispersion interval for 500 simulations).

^§^ Truly associated genes are more frequent in GS’ than in GS.

To complete each simulation run, we needed to perform the following steps:

**Table:** 

MA step:	simulate marker association
GWAS step:	perform GWAS
GSA step:	perform GSA
META-GSA step:	perform META-GSA and SPP

For the **MA step** we considered samples of 500 cases and 500 controls. The minor allele frequencies of all markers were set to 30%. The distribution of the genotypes was assumed to be in Hardy-Weinberg Equilibrium (HWE). The prevalence of a binary phenotype in the population was set to 5%. The relative risk was pre-specified and ranged between 1.0 and 1.5. The direction of an association was chosen randomly, assuming both directions as being equally likely.

In the **GWAS step** we considered 100 genes with one genotyped marker each. For each of these 100 markers, we performed a two-sided Armitage trend test, yielding *p*_*m*,*s*_-values and fitted log-additive models to determine the direction of observed association (*d*_*m*,*s*_ = +*1 if OR*≥*1* and *d*_*m*,*s*_ = -*1 if OR*<1). Markers strongly deviating from HWE in controls (*p*_*m*,*s*_ *<1×10* ^*−7*^) were excluded from further analysis.

In the **GSA step,** we applied a one-sided Wilcoxon’s rank-sum test [19] to investigate enrichment of low *p*_*m*,*s*_-value in *GS* (yielding *p*_*GS*,*s*_). The gene set *GS* under investigation always consisted of 10 genes, the remaining 90 genes were considered as *GS’*.

In the **META-GSA step,** marker-specific *p*_*m*,*s*_-values and directions *d*_*m*,*s*_ were subsequently combined to PDR-values. Finally, we determined gene-set-specific *p*_*GS*,*s*_-values. There was no need to correct for multiple testing because we only regarded a single *GS*.

Subsequently and for the purpose of comparison, we simply summarized *p*_*GS*,*s*_-values applying Fisher’s inverse χ^2^-method to *p*_*SPP*_-values.

As an alternative approach, one may consider a so-called mega-analysis (individual participant data meta-analysis) directly based on all participants’ geno- and phenotype data, if or when available, as gold standard. Advantages of such an approach would be the use of only one statistical model throughout the analysis, the inclusion of available unpublished data, a larger sample size that allows subset analysis, the avoidance of ecological bias, and potentially higher power [20, 21]. However, it has been demonstrated mathematically that summarizing summary results, i.e. performing a meta-analysis for main effects of each marker, is as efficient as pooling genotype data, i.e. performing a mega-analysis for each marker, if no covariates are considered [22]. This is similarly true for investigations of gene x environment interactions [23]. In addition, limitations associated with mega-analysis need to be dealt with, such as ethics or confidentiality constraints in sharing study data, comparable data quality and completeness, or sufficient system capacity for storage and transfer. Analyzing studies separately and summarizing their results in a meta-analysis also enables adjustment for different sets of covariates for different studies, as well as allowing for heterogeneous genetic effects between studies [24].

Since the single marker associations of all studies are available (which is a prerequisite for META-GSA), we also accomplished a random effects meta-analysis for each marker and then performed a single GSA (denoted as pooledGWAS-GSA) for the purpose of comparison with META-GSA and SPP. Thus the combining of studies is switched to the level of markers, respectively genes. This approach is located halfway between an individual participant data meta-analysis and an aggregate study-level data meta-analysis.

We considered 500 simulations for each scenario. The percentages of *p*_*META-GSA*_-values, *p*_*pooledGWAS-GSA*_-values or *p*_*SSP*_-values lower than the level of significance of 5% were used as power for the respective test. Given a true type I error of 5%, the observed type I error may range from 3% to 7% (95% random dispersion interval for 500 simulations).

An overview of the scenarios, the achieved type I error, and power is presented in Table 2.

### Type I error

As expected, in about 5% of the simulations, a *GS* is falsely discovered when the pattern of association does not differ between *GS* and *GS’* (scenarios 1–3). Any observed type I error is within random variation according to the number of simulations. However, in the case of mixed structures with risk markers in *GS* and *GS’* (scenario 20), SPP, pooledGWAS-GSA and META-GSA seem to be conservative (type I error ranges between 2.6% and 4.8%).

**Fig 2:**
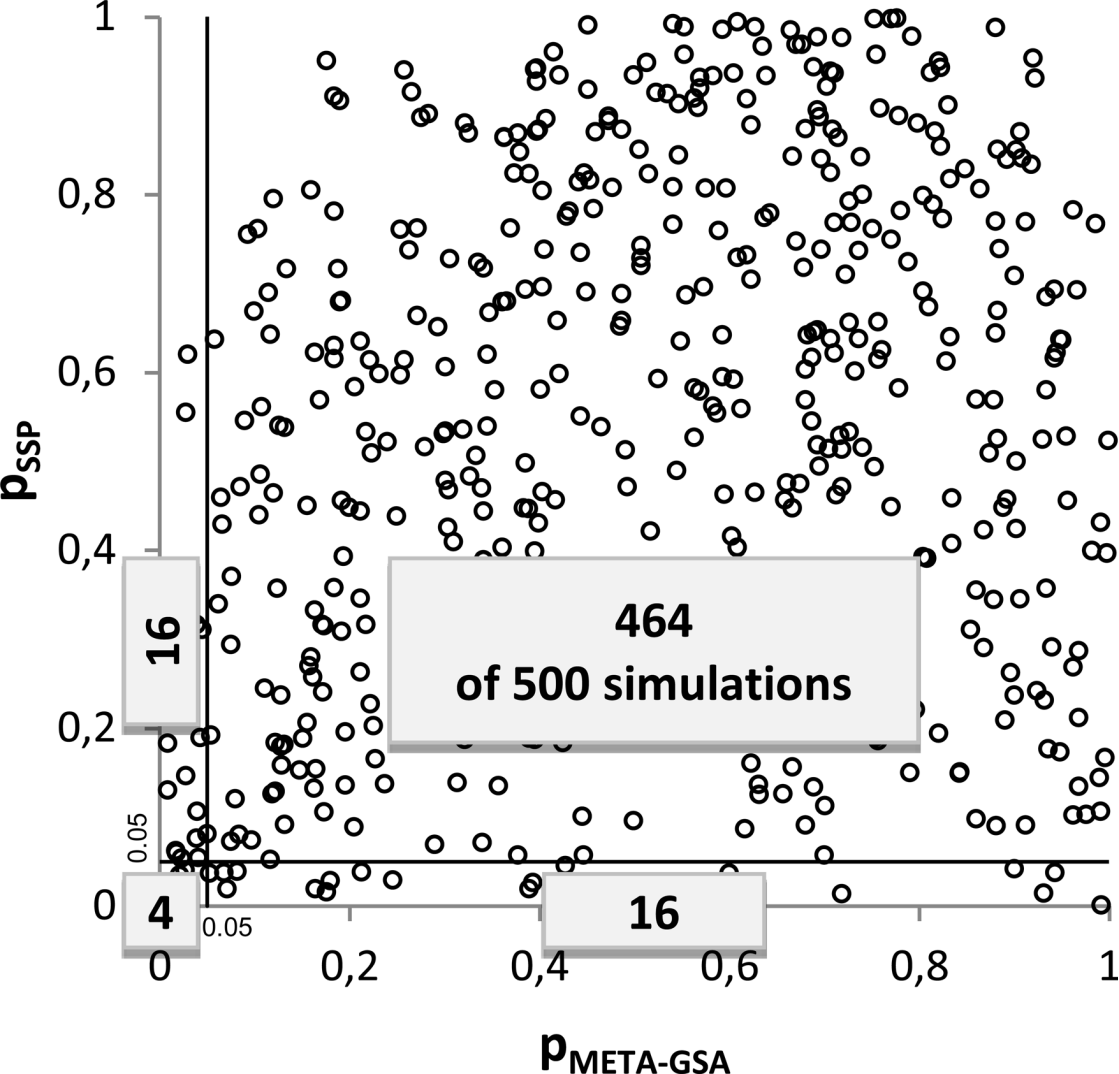
Correlation of *p*_*META-GSA*_ and *p*_*SPP*_ in scenario no. 1. The numbers of simulations out of a total of 500 are depicted. Gene sets are classified as significant (p ≤ 0.05) or not significant using SPP and META-GSA.

Interestingly, there seems to be almost no systematic overlap in discovered *GSs* between META-GSA and SPP. For example, in scenario 1 the *GS* was falsely discovered in 20 simulation runs by each approach. However, *GS* was concordantly discovered by META-GSA and SPP in only four runs (see Table 3). Furthermore, almost no correlation between *p*_*META-GSA*_ and *p*_*SPP*_ was observed (see Fig 2). False-positive gene sets found by META-GSA and SPP only partially overlap. We assume that this may result at least in part from respecting the *concordance of association patterns*.

**Table 3:** Comparing the number of significant findings of META-GSA with SPP in selected scenarios.

	patterns of true marker-phenotype associations		META-GSA*	SPP	
Scenario No.	*RRs in GS of interest*	*RRs in complementary GS´*		*p≤5%*	*p>5%*.
**H**_**0**_ **: F(RR|GS) = F(RR|GS´)**					
1	10x1	90x1	*p≤5%*	4	16
			*p>5%*	16	464
2	10x1.2	90x1.2	*p≤5%*	6	24
			*p>5%*	18	448
20	10x1.5	90x1.5	*p≤5%*	13	2
			*p>5%*	34	451
**H**_**A**_ **: F(RR|GS)≠F(RR|GS´)**					
4	5x1 5x1.1	90x1	*p≤5%*	7	39
			*p>5%*	20	434
5	5x1 5x1.2	90x1	*p≤5%*	38	94
			*p>5%*	9	359
6	5x1 5x1.3	90x1	*p≤5%*	103	141
			*p>5%*	2	254
7	5x1 5x1.4	90x1	*p≤5%*	148	142
			*p>5%*	—	210
8	5x1 5x1.5	90x1	*p≤5%*	184	110
			*p>5%*	—	206
17	5x1 4x1.2 1x1.5	90x1	*p≤5%*	77	101
			*p>5%*	8	314
18	5x1 4x1.2 1x1.5	63x1 18x1.2 9x1.5	*p≤5%*	27	2
			*p>5%*	28	443
19	5x1 4x1.2 1x1.5	45x1 36x1.2 9x1.5	*p≤5%*	7	1
			*p>5%*	35	457

* Applying Kendall’s correlation coefficient

### Power

Next, we consider those scenarios in which patterns of association differ between *GS* and *GS’* (scenarios 4–19). The power of a META-GSA exceeded that of SPP in all considered scenarios, that of a pooledGWAS-GSA was always in between. Only in the presence of stronger marker-specific associations (e.g. true RR = 1.5) and a larger number of studies (scenarios 8, 15, and 16) were pooledGWAS-GSA and META-GSA comparable in terms of power.

**Fig 3:**
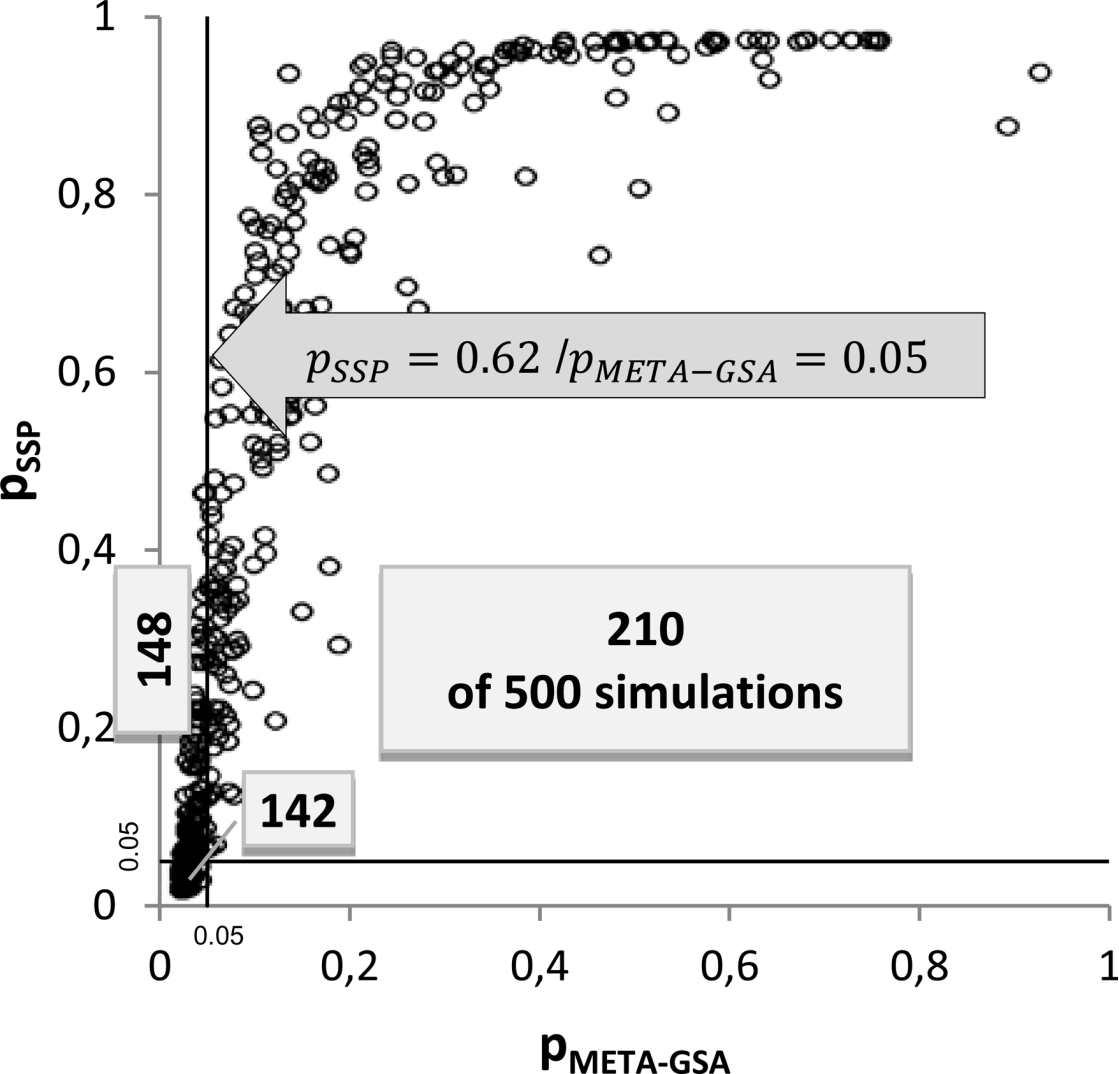
Correlation of *p*_*META-GSA*_ and *p*_*SPP*_ in scenario no. 8. The numbers of simulations out of a total of 500 are depicted. Gene sets are classified as significant (p ≤ 0.05) or not significant using SPP and META-GSA.

For example, the simulated power of META-GSA was almost 60% if five markers with a true RR = 1.5 belong to *GS* (scenario 8). For SPP, we achieved a power of only 37% and a power of 56% for a pooledGWAS-GSA in the same scenario. The *GS* was identified by META-GSA in 294 out of 500 runs, while SPP identified the *GS* in only 184 of those 294 runs (approximately two out of three runs). In every simulation run in which META-GSA failed to identify the *GS* correctly, SPP also failed (see Table 3). Using SPP, the largest p-value found in these 294 runs was *p*_*SPP*_ = *0*.*62*, indicating that gene sets are missed by SPP, which were identified by META-GSA (see Fig 3).

For the pattern combination of scenario 8, we also simulated the power dependent on the number of studies to be combined (scenarios 9–16) and found a steady increase in power for all three approaches. META-GSA always proved superior to both SPP and pooledGWAS-GSA.

Regarding scenarios 4 to 8, we found that the more *GS* differs from *GS’*, the larger the benefit becomes in terms of power of META-GSA compared to SPP.

Finally, we simulated three patterns with varying associations in both gene sets *GS* and *GS’* (scenarios 17–19). Note that a third to a half of all the genes in *GS’* were set to be associated, which is an extremely and unrealistically high proportion. We found a remarkable loss of power if the *GS* of interest is dominated by other gene sets in *GS’*, meaning that truly associated genes are more frequent in *GS’* than in *GS* (scenario 19). The power of a META-GSA even falls below the level of significance, as was the case for a pooledGWAS-GSA. This was to be expected, since we simulated according to the “competitive hypothesis”. SPP behaves better in such a scenario. However, a demonstrated power of 8% is far from promising though.

## Application: GO-Pathways and Lung Cancer

### Previous comparison of GSA methods

Data from four case-control GWASs of lung cancer risk were previously implemented [15] to compare the performance of four different GSA approaches: EASE [3], GenGen (developed from GSEA) [8], SLAT [16], and mSUMSTAT [15] a modification of the SUMSTAT approach [25]. The lung cancer cases and controls were taken from the Central Europe Study [26], a study of the Lunenfeld-Taneberg Research Institute / Toronto [26], the German Lung Cancer Study [27, 28], and a study of the MD Anderson Cancer Center / Texas (MDACC Study, non-small cell lung cancer cases and controls) [29]. This research was granted a renewed protocol approval from the Dartmouth Committee for Protection of Human Subjects on 7/30/2014 with id STUDY00023602. The specific protocol for this research covers meta-analysis of data from several studies for which participants previously signed consent documents and for which genome-wide association studies have already been completed. The current research covers the combination of data across studies. These studies are shared amongst members of the Transdisciplinary Research in Cancer of the Lung / International Lung Cancer Consortium (TRICL / ILCCO). Data from these four studies were combined into two data sets: 1) Central Europe and Toronto (CETO); and 2) Germany and MDACC (GRMD), in order to create adequate sample sizes and statistical power to detect associations in the pathway analyses. More details on the studies are provided by Fehringer et al. [15].

In summary, the genotypic information from 8,542 individuals (3,897 cases and 4,646 controls) was used. Genotyping of more than 300,000 SNPs assigned to about 18,000 genes was performed using either the Illumina HumanHap300 or HumanHap550 chips. In total, 7,163 genes were assigned to at least one of 421 GO level 4 pathways (obtained from the GenGen website), comprising 15 to 200 genes to avoid testing overly large or small gene sets. Only a single gene set (GO0015464: acetylcholine receptor activity) was found nominally significant in both data sets by two of the four GSA methods. Some biological interpretation of how acetylcholine receptor activity can influence the risk for lung cancer was given [15].

The resulting files, containing p-values according to gene set and method as well as SNP-to-gene assignment, gene-to-pathway assignment, and SNP-wise association, were the same as used previously. The only information needed which had not been used previously was the LD measure between markers within genes to homogenize PDRs. These quantities were therefore estimated based on a sample of 484 Caucasians used as controls in the German Lung Cancer Study. All controls were genotyped for 561,466 SNPs using the Illumina Sentrix HumanHap550 Beadchip. How we corrected for multiple testing is outlined in S6 Text.

## Results

At the nominal level of significance of α = 5%, 30 out of all 421 gene sets were found to be significant according to *p*_*permut*_ by at least one of the GSA methods (EASE, GenGen, SLAT, mSUMSTAT), when only the most significant marker was selected to determine concordance of the direction of association. Remarkably, 26 gene sets thereof were identified irrespective of which GSA approach was chosen. In contrast, 134 pathways would have been identified as nominally significant by at least one of the GSA methods when using SPP and only 8 by all of the methods. This impressively demonstrates the selective character of META-GSA. Moreover, a strong variation in the number of nominally significant gene sets between GSA methods was observed when analyzed by SPP (28 to 72 gene sets). This maybe can be attributed to the principles of the applied GSA methods. EASE or GeneGen are based on the count of genes classified as *significant* or *not significant*. Analyzed by SPP the number of nominal significant gene sets are 32 or 28, respectively. SLAT an mSUMSTAT make use of the magnitude of single marker significance (p-values or χ^2^-values). Analyzed by SPP the number of nominal significant gene sets are 89 or 72, respectively.

Applying META-GSA, results were consistent irrespective of which GSA method was used. Using all SNPs at a gene, 46 to 47 gene sets were nominally significant; using only significant SNPs at each gene, 22 to 24 gene sets appeared nominally significant. Using the most significant SNPs for each gene, the number of significant gene sets lay in between (27 to 30 gene sets, see Table 4). This demonstrates that META-GSA substantially reduces the influence of the GSA method applied on the final results. On the contrary, the dependency of SPP on the GSA method applied can clearly be demonstrated for GO0015291, for which *p*_*SPP*_, adjusted for multiple testing ranges, from 0.0003 to 1.0. Further details demonstrating the inhomogeneity between GSA-methods and the homogenizing effect of META-GSA are given in S7 Text.

**Table 4:** META-GSA vs. SPP: Number of nominally significant gene sets by GSA methods.

	*META-GSA (p*_*permut*_ *<0*.*05)*			*SPP*
*GSA approach*	*all SNPs*	*all significant SNPs*	*most significant SNP*	(*p*_*SPP*_ *≤0*.*05*)
***EASE***	46	22	27	32
***GenGen***	46	24	29	28
***SLAT***	46	23	28	89
***mSUMSTAT***	47	23	30	72

The homogenizing effect of META-GSA can also be demonstrated by the strong pairwise rank correlations between the *p*_*META-GSA*_-values of the GSA-methods, when using the most significant SNP to determine concordance (ρ > 0.85) in contrast with only moderate correlations regarding *p*_*SPP*_-values (ρ ≈ 0.5; see Table 5).

**Table 5:** Rank correlation of p-values comparing GSA methods (best SNP approach).

	*META-GSA*			*SPP*		
	*GenGen*	*SLAT*	*mSUMSTAT*	*GenGen*	*SLAT*	*mSUMSTAT*
***EASE***	0.93	0.88	0.93	0.54	0.40	0.55
***GenGen***		0.90	0.95		0.52	0.73
***SLAT***			0.90			0.70
***mSUMSTAT***						

A comparison here with pooledGWAS-GSA cannot be made, since gene set analyses were previously applied to the presented data only for each study, but not for a pooled GWAS.

Using all markers allocated to a gene to determine concordance and applying the GSA approach EASE revealed GO-pathway GO0015291 (“transmembrane transporter activity”) to be significantly enriched with associated genes (*p*_*permut*_ = 0.0001; *p*_*META_GSA*_ = *0*.*315*). When GSA was performed using another method, significance was reached nominally (*p*_*permut*_ ≤ 0.001) but not when adjusted for multiple testing (*p*_*META_GSA*_ ≈ *0*.*2*). In comparison with META-GSA, even nominal significance was missed applying SPP. For the previously identified gene set GO0015464 [15], we achieved almost the same nominal p_permut_-values as for GO0015291 and across GSA methods.

## Discussion

It is increasingly recognized that GSA can extend GWAS approaches by incorporating existing knowledge of biological processes, with the aim of identifying disease-related pathways. GSA has gained great popularity and several approaches have been proposed. Although the pros and cons have been discussed [9, 19] and points to improve have been formulated [30], it has only been mentioned that there is a need to replicate pathway association findings to avoid false positive results [30]. According to our knowledge, there is no formal method to combine the results of several GSAs. The basic criticism on applying simple p-pooling (SPP) is a lack in interpretability of results when single markers differ in their attributed role as risk or predictive factor. Here we propose the quantitative approach META-GSA to combine such results, respectively *GS-significance*, by incorporating *concordance* of single-marker *association patterns* between studies, relevant for the *GS* of interest.

The main steps of META-GSA are first to determine the *concordance of association patterns*; second, to use these to derive a weight for each study; and third, to apply a weighted version of Fisher’s inverse χ^2^-method to summarize significance of GSAs in a single meta-analytical p-value. Thus *significance* of GSA-results and *concordance* of single-marker association are combined. META-GSA can be further considered as a conditional approach, testing *GS-significance* conditional to, or in the presence of *concordance of association patterns*.

Since the mathematical conditions of the weighted version of Fisher’s inverse χ^2^-method are not fulfilled, the application of a CPU-intensive permutation procedure is required. Thus we investigated the effort and benefits of META-GSA in comparison with SPP, which is fast but does not address *concordance of association patterns* in any way. Both methods keep type 1 error at the specified level. However, under H_0_ the results of META-GSA and SPP were found to be almost uncorrelated. We believe this to be down to addressing or ignorance of the *concordance of association patterns*, respectively. Moreover, META-GSA was found to be more powerful than SPP. The greater the number of studies combined, the larger the advantages in power became. The gain in power can be clearly attributed to the fact that META-GSA is more efficient at separating the “wheat from the chaff” for gene sets in terms of false replicate findings when *concordance of association patterns* is not given. Given a unique truly associated gene set, META-GSA successfully yielded a significant result twice out of 3 times, whereas SPP failed to find even one of these two significant results.

We also compared META-GSA to a pooledGWAS-GSA approach. For the latter, the combining of studies is switched to the level of markers, followed by a single GSA performed on the pooled marker-specific associations. In general, we found META-GSA to outperform pooledGWAS-GSA. This is similar to the fact that a mega-analysis does not outperform a meta-analysis when testing for single marker association [22]. One further advantage of META-GSA is the fact that heterogeneity in the strength of association for single markers or genes, respectively, between studies does not necessarily cause lower power, as long as other genes belonging to *GS* compensate such deficiency. “Between-study heterogeneity … can offer valuables insight for further clarification of gene-disease associations” [31].

Furthermore, META-GSA is applicable to any GSA method selected, even those using individual participants’ genotype data, which may prove to be more suitable and more powerful than methods based on GWAS summary results (pooledGWAS-GSA) [2, 32]. Resting the GSA on common effect estimates can become critical in the case of strong study heterogeneity for few or many markers, since the existence of a common marker-specific association in such a situation is doubtful. For META-GSA, study heterogeneity results in low concordance of the patterns of study-specific association estimates and subsequently in low study weights, which simply reduces the power, however without violating such a critical assumption.

Nevertheless, the simulations were configured to compare a gene set of interest to all other genes. If the investigated gene set is truly dominated by other, containing more strongly associated genes, the simulation revealed META-GSA as performing poorly, although META-GSA is not based on the so-called “competitive hypothesis”. This disadvantage is related to the genetic architecture of complex diseases that always needs to be taken into account when performing pathway analysis [1, 9]. GSA is likely to be informative if the interplay of hundreds of genes in pathways contributes to the susceptibility to a disease or trait. In the presence of a single, strongly associated gene, GSA methods may be of less interest to research that addresses the “competitive hypothesis”. However, such a drawback can be obviated by e.g. excluding such genes and other genes of already identified pathways from the analysis. GSA methods addressing the “self-contained hypothesis” are said to be more appropriate, as is the case in studies limited to candidate genes. Note that META-GSA is applicable to both types of hypotheses underlying a GSA. META-GSA, however, has only limited use for pleiotropic investigation, since concordance of gene effects between all studies is regarded as important. This matters when considering only one outcome entity. Summarizing genetic associations with distinct but putatively related traits, one may allow susceptibility loci to be associated in subsets or in different directions for different traits [33].

Applying META-GSA to (only) two independent GWAS/GSA investigations into lung cancer revealed that META-GSA is more robust than SPP compared to the previously used GSA method. Furthermore, we discovered a positive relationship between the sufficiency in condensing single-SNP information to a gene-level statistic and the likelihood of identifying a related gene set. Nevertheless, this observation is related to the general discussion of how to condense SNP information within a gene, addressing LD structures, gene size and length, overlapping genes, or the statistical method applied [1, 9, 30].

All the same, META-GSA has some critical points that need to be mentioned. First, it is necessary to estimate LD between neighboring markers to be able to calculate PDRs of differing markers across studies. However, patterns of LD in the human genome are said to be noisy, they vary from region to region, and are difficult to determine for broadly defined ethnicities such as Caucasians, Africans, and Asians. For example, LD in non-African populations extends over longer genomic distances than in Africans [34]. Since it is known that LD-patterns in human subpopulations are different [35], this can be problematic if one aims to combine results across different ethnicities or if the source population of a study is known to be admixed. To circumvent the problem of multiple ethnicities, we considered only studies of Caucasians in our analysis.

Secondly, a number of alternative ways to combine marker-level estimates to a gene-level statistic have already been published. This includes the inverse gamma method (GM)[36], the rank truncated product method (RTP)[37], the truncated product method (TPM)[36]; the adaptive rank truncated product method (ARTP)[38], or any approach for that matter, in which the number of true null hypotheses [39] is estimated. Any of these methods could have been implemented instead. The pros and cons of several methods have been discussed elsewhere [36–39].

Thirdly, the use of a permutation procedure is time and CPU-intensive. We therefore developed a “quick but dirty” test to avoid the computational burden caused by unnecessary permutations. This test consists of a marginal combination of significance and *concordance of association patterns* (details are given in S8 Text). However, we must recognize that the ability to sort out only “bad candidate gene sets” for the permutation was fairly limited. That said, we could speed up the program by implementing early-stopping rules. That again also means that the resulting p-values are insufficiently approximated and imprecise, in particular for gene sets clearly not significant.

Fourthly, p-values are the only results. As with most GSA approaches META-GSA does not deliver any effect estimation.

Fifthly, gene-set analysis results are often prone to sources of bias including a) unequal gene set size, a) LD patterns and c) overlapping genes [6, 30, 40]. To be robust against a) and b) multiple comparison procedures, such as Sidak's correction [6]; decorrelation tests [5]; or omnibus tests, such as Fisher’s inverse Chi^2^-method (e.g. SLAT [16]) are used. To be robust against a), b) and c) permutation procedures (e.g. ALIGATOR [41]) or single model approaches comprising all SNPs allocated to a gene-set and correcting for the number of effective markers (e.g. kernel methods[32]) can be used. An additional source of bias results from d) low resolution knowledge bases. To perform GWASs and GSAs one needs to annotate SNPs to genes which are annotated to pathways in a static way, referring to public databases. In contrast, genes act dynamically and may have for instance several transcripts which can be active or passive in a certain pathway. Thus, a static annotation maybe doesn’t matches well to a dynamic biological process. Furthermore, e) both SNP-to-gene as gene-to-pathway annotation databases reflect at the best the state of knowledge and should therefore considered as incomplete (to an unknown degree). Because GSA methods, as they have been proposed, use genomewide genotype data therefore are f) unable to model dynamic response of a gene in the course of disease development. In the same they are g) limited to model response to external stimuli [40]. These additional sources of bias (d-g) affect all GSA approaches. Hence, META-GSA is concerned (biased) to the same extend as the GSA approaches aimed to be combined.

All in all, we demonstrate that META-GSA may be a powerful add-on tool in the research of the genetic architecture of complex traits or diseases. One can attribute its benefit to the incorporation of the concordance of single-marker association into the test statistics.

All programs were implemented in SAS 9.3 (SAS Institute, NC, USA) and are provided as S11 Text.

## Supporting Information

S1 Fig Graphical representation of Fisher’s p-pooling method for independent and identical target measures. The test statistic *M* is the *sum of -2ln(ps) for s = 1 to n*_*s*_ *studies*, which follows a χ^2^-distribution, assuming all tests point towards the same (common) direction (identical target measures). Note: the lower the p-value, the higher the statistical evidence and the longer the arrow.
(TIF)

S2 Fig Graphical representation of Makambi’s weighted p-pooling method for different, hence imperfect correlated target measures. The test statistic *M* is the *sum of -2w*_*s*_ *ln(p*_*s*_ *) for s = 1 to n*_*s*_ *studies*, which follows a χ^2^-distribution, allowing for some deviations in the direction of the target measures. No study points towards a common direction.
(TIF)

S1 Text Details Chi^2^ method. (DOCX)

S2 Text Alternative PDRs. (DOCX)

S3 Text Between marker LD. (DOCX)

S4 Text Significance testing. (DOCX)

S5 Text Speeding up the permutation. (DOCX)

S6 Text Correction for multiple testing. (DOCX)

S7 Text Comparison META-GSA with SPP. (DOCX)

S8 Text A quick but dirty test. (DOCX)

S9 Text PRISMA Checklist. (DOCX)

S10 Text PRISMA flow diagram. (DOCX)

S11 Text META-GSA routines. (7Z)
