# Supplementary figures and images for: META-GSA: Combining Findings from Gene-Set Analyses across Several Genome-Wide Association Studies
Source: PLoS One. 2015 Oct 26;10(10):e0140179. doi: 10.1371/journal.pone.0140179 (PMC4621033; doi:10.1371/journal.pone.0140179)

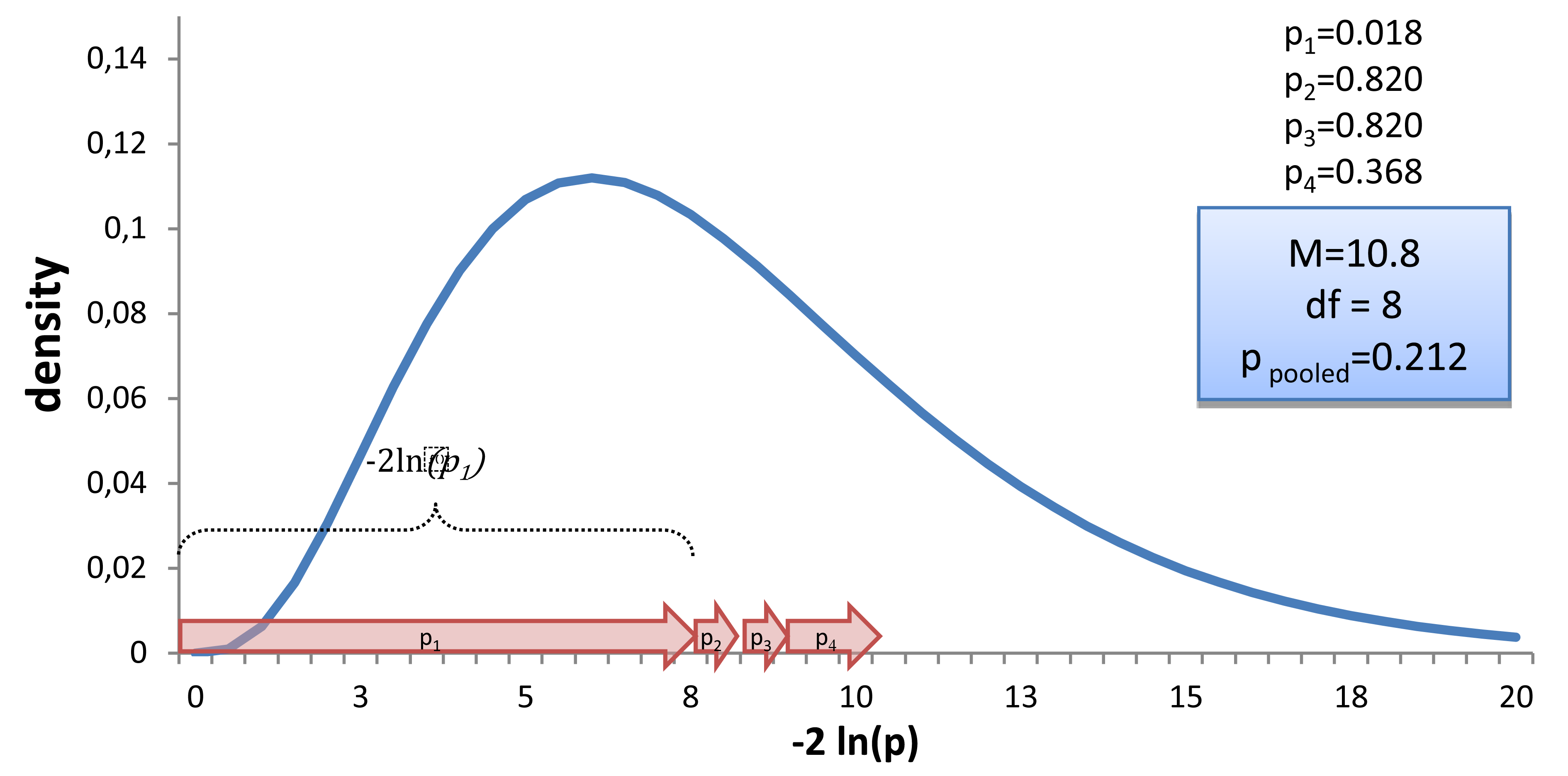

Supplement: S1 Fig — The test statistic M is the sum of -2ln(ps) for s = 1 to n s studies, which follows a χ2-distribution, assuming all tests point towards the same (common) direction (identical target measures). Note: the lower the p-value, the higher the statistical evidence and the longer the arrow. (TIF) [file pone.0140179.s001.tif]

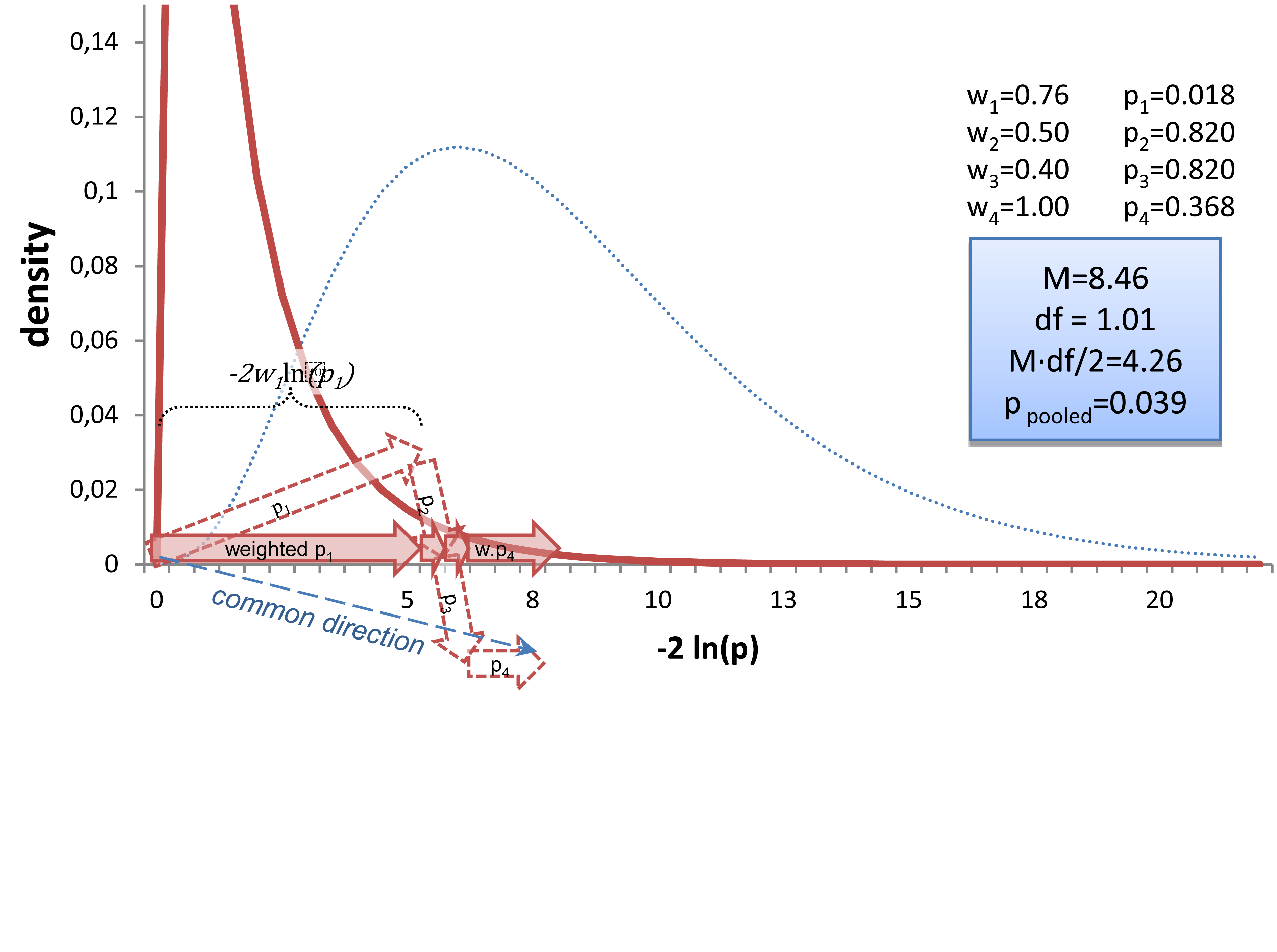

Supplement: S2 Fig — The test statistic M is the sum of -2w s ln(p s ) for s = 1 to n s studies, which follows a χ2-distribution, allowing for some deviations in the direction of the target measures. No study points towards a common direction. (TIF) [file pone.0140179.s002.tif]
