# Supplementary material for: META-GSA: Combining Findings from Gene-Set Analyses across Several Genome-Wide Association Studies
Source: PLoS One. 2015 Oct 26;10(10):e0140179. doi: 10.1371/journal.pone.0140179 (PMC4621033; doi:10.1371/journal.pone.0140179)
Supplement: S1 Text — (DOCX) [file pone.0140179.s003.docx]

## Details to **Makambi’s enhancement and Patnaik Type χ²-approximations of Fisher’s inverse χ²-method**

***Fisher’s inverse χ²-method***

**Usually the** p-value *p* of a statistical test (apart from exact tests) follows a uniform distribution, if H_0_ is true. Consequently $-2ln(p)$ follows a χ²-distribution with two degrees of freedom (df). The sum $M_{n_{s}}=-2\sum_{s=1}^{n_{s}} \ln(p_{s})$ of *n_s_* independent tests follows again a χ²-distribution, with *df=2n_s_* degrees of freedom. However, $M_{n_{s}}$is meaningful if and only if all related test statistics *T_s_* point towards the same direction in terms of target measure.

***Makambi’s enhancement, Patnaik Type χ²-approximations***

Makambi enhanced [[1](#_ENREF_1)] this method by introducing pre-specified weights *w_s_* for each study
($\sum_{s=1}^{n_{s}} w_{s}=1$ and $w_{s}>0$ for all *s*) and correlation coefficients $\rho_{s,s´}$, when different target measures are tested, which are by nature imperfectly correlated (e.g. odds ratios for incidence or mortality or differences in prevalence). He reformulated the test statistic to $M_{n_{s}}=-2\sum_{s=1}^{n_{s}} w_{s}\ln(p_{s})$. He argued that, under *H_0_*, the term $\frac{vM_{n_{s}}}{E(M_{n_{s}})}$ is approximately χ²-distributed with *df=ν*, where $v=2\frac{\left\{ E\left( M_{n_{s}} \right) \right\}^{2}}{var\left( M_{n_{s}} \right)}$, as given by Patnaik [[2](#_ENREF_2)]. Makambi further demonstrated that $E(M_{n_{s}})\equiv2$, so that $v=\frac{8}{\mathrm{var}(M_{n_{s}})}$. The variance of $M_{n_{s}}$ is a function of the weights *w_s_* and the pairwise correlation coefficients *ρ_s,s´_*, and given as ${var(M}_{n_{s}})=4\sum_{s=1}^{n_{s}} w_{s}^{2}+\sum_{s´=1}^{n_{s}} \sum_{s\neq s'=1}^{n_{s}} w_{s}w_{s'}\left( 3.25\rho_{s ,s´}+0.75\rho_{s,s´}^{2} \right)$, with $s=s': \rho_{s,s´}=1$ and $s\neq s': 0\leq\rho_{s,s´}\leq1$. Although the correlation coefficient *ρ_s,s´_* is valid within the range $-1\leq\rho_{s,s'}\leq+1$, only positive values need to be regarded. Otherwise, pooling p-values to increase evidence does not make any sense. Subsequently, the overall *H_0_* should be rejected at a significance level α if $M_{n_{s}}\geq2v^{-1}\chi_{v,1-\alpha}^{2}$. If all target measures are the same, e.g. all are odds ratios as regarded for the power simulation of META-GSA, then the correlation coefficient will be $\rho_{s,s´}=1$.

Graphical representations of Fisher’s p-pooling method and Makambi’s weighted p-pooling method are given in Supplementary Figure 1 and 2. One may understand that achieving some summary evidence through pooling p-values is meaningful only if the sense of the association is the same (advantageous/protection or disadvantageous/risk). P-values may be regarded as “arrows pointing towards evidence”. The above-mentioned sense of association is then equal to the direction of these arrows. Assigning a lower weight to a study is equivalent to turning the “arrow” towards a common direction, so that only a part of the observed p-value is counted.

Supplementary Figure 1: Graphical representation of Fisher’s p-pooling method for independent and identical target measures


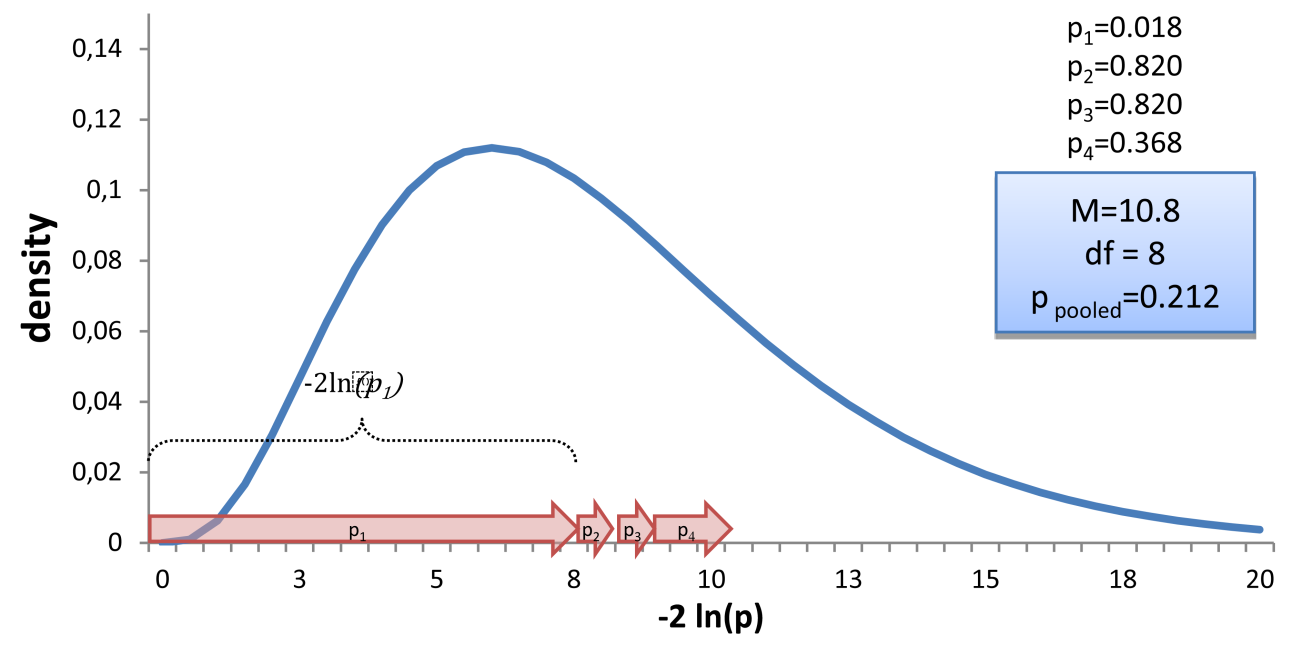


The test statistic *M* is the *sum of -2ln(ps)* *for s=1 to n_s_ studies*, which follows a χ²-distribution, assuming all tests point towards the same (common) direction (identical target measures). Note: the lower the p-value, the higher the statistical evidence and the longer the arrow.

Supplementary Figure 2: Graphical representation of Makambi’s weighted p-pooling method for different, hence imperfect correlated target measures


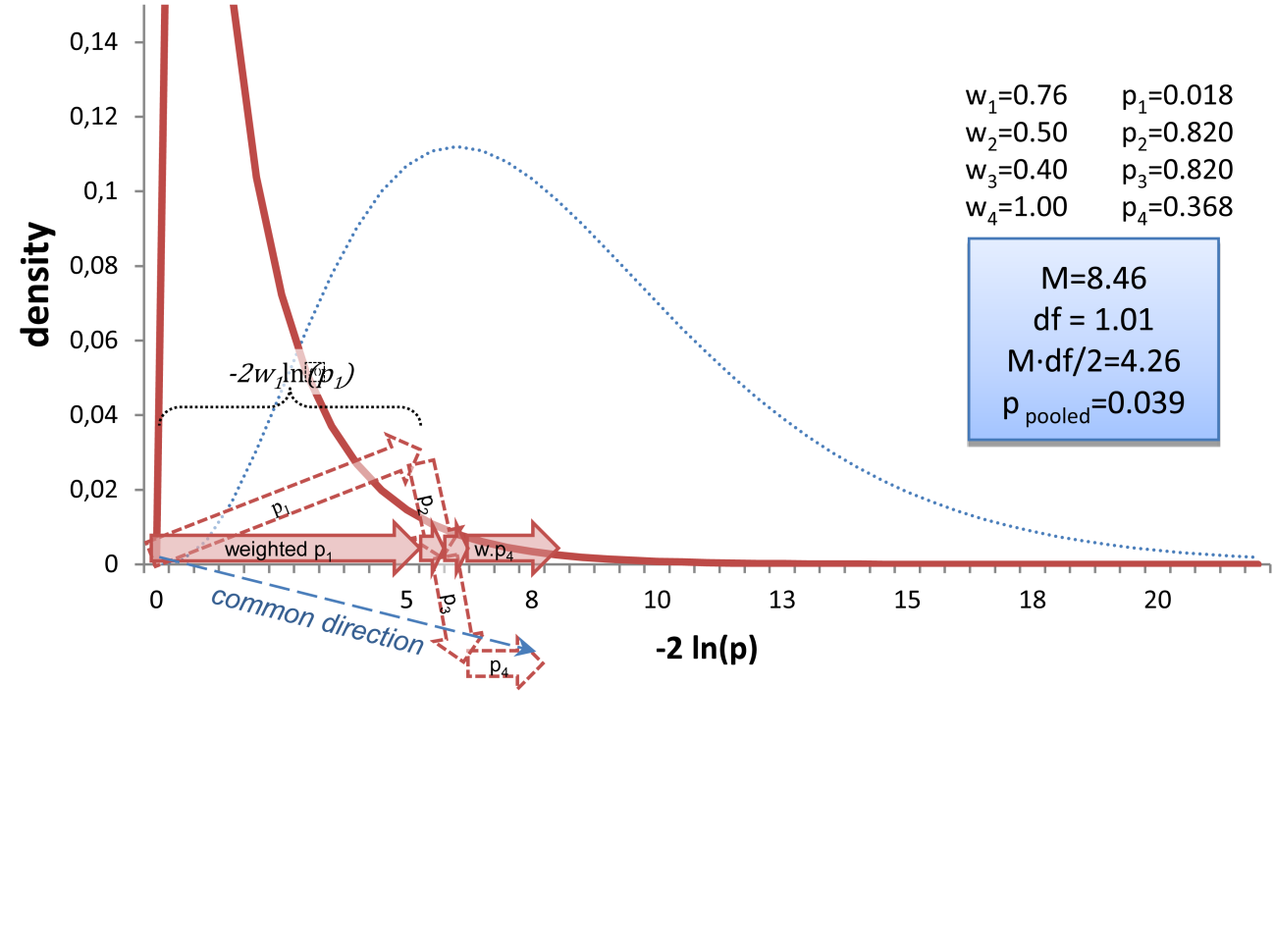


The test statistic *M* is the *sum of* *-2w_s_ln(p_s_) for s=1 to n_s_ studies*, which follows a χ²-distribution, allowing for some deviations in the direction of the target measures. No study points towards a common direction.

Reference

1. Makambi K (2003) Weighted inverse chi-square method for correlated significance tests. Journal of Applied Statistics 30: 225-234.

2. Patnaik PB (1949) The non-central chi²- and F-distribution and their applications. Biometrika 36: 202-232.
