# Supplementary material for: META-GSA: Combining Findings from Gene-Set Analyses across Several Genome-Wide Association Studies
Source: PLoS One. 2015 Oct 26;10(10):e0140179. doi: 10.1371/journal.pone.0140179 (PMC4621033; doi:10.1371/journal.pone.0140179)
Supplement: S2 Text — (DOCX) [file pone.0140179.s004.docx]

## Alternative definitions of directed reverse p-values

To combine significance and direction of an observed marker-specific association in a manner similar to a correlation coefficient, such that zero represents no evidence and the limits +1 and ‑1 represent strong evidence of positive or negative association. Thus, we defineed a

- directed reverse p (DRP) as $p'_{m,s}=d_{m,s}\cdot(1-p_{m,s})$ (S1)

Alternatively one can define:

- directed odds p (DOP) $p'_{m,s}=d_{m,s}\cdot(p_{m,s}^{-1}-1)$ (S2)

Because $p_{m,s}^{-1}-1=\frac{1-p_{m,s}}{p_{m,s}}$, DOP can be considered as the directed odds of p.

- directed squared root inverse p (DSQP) $p'_{m,s}=d_{m,s}\cdot\sqrt{p_{m,s}^{-1}-1}$ (S3)

where $d_{m,s}=sign\left( \theta_{m,s} \right)$ $\in\left\{ -1,+1 \right\}$ (S4)

All three are monotone transformations of p-values, but (S2) DOP and (S3) DSQP are not applicable if *p_m,s_ ≡*0, which is never the case. However, small p-values which are related to very large values of PDR, might be inexact to a certain degree due to computational difficulties in approximating the extreme tails of any null distribution. If *p_m,s_=1*, all three optional functions take on the value 0. The behavior of these options is portrayed in Supplementary Figure 3. Applying (S2) DOP or (S3) DSQP would clearly shift relatively more weight to genes with low p-values, while applying (S1) DRP assigns a substantial weight to genes showing moderate significance.

Supplementary Figure 3: Three options for *directed reverse p-values* (PDR)


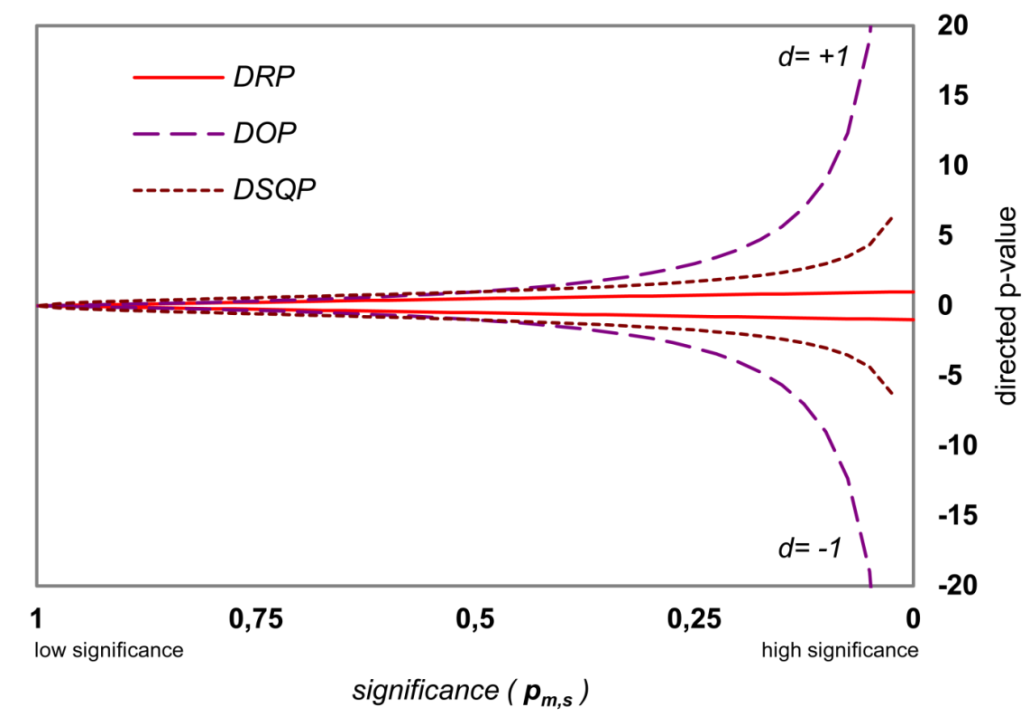


directed reverse p (DRP); directed inverse p (DOP); directed squared root inverse p (DSQP)

### Extended tables for type 1 error and power

Regarding $p'_{g,s}$as a function of the true association $\theta_{m,s}$, the sample size *n_m,s_*, perhaps population stratification and the fitted statistical model, PDRs might be “scaled” differently between studies. Hence, in assessing the concordance between PDR-profiles to determine study weights, rank correlation (e.g. Spearman’s ρ or Kendall’s τ) seems advantageous over Pearson’s correlation, because one neither needs to assume homogeneity of such influencing factors across studies nor any kind of linearity.

To show this, we compared the type I error and power of META-GSA, when applying different definitions of PDR in combination with different method to assess the correlation between PDR-profile (Kendall, Spearman, Pearson). When using a rank-correlation methods, the definition of PDR get regardless, because there are isotone transformations of each other.

Calculating PDR according equation (S1) and applying rank-correlation outperformed all other possibilities in terms of power (see Supplementary Table 1). Even the choice of the rank-correlation method (Spearman’s ρ or Kendall’s τ) seems to be regardless.

Supplementary Table 1: Power of META-GSA, pooledGWA-GSA and SPP across all studies, based on 100 genes with 1 marker each

| patterns of true marker-phenotype associations | | | | *no.  studies* | META-GSA | | | | |
| --- | --- | --- | --- | --- | --- | --- | --- | --- | --- |
| *scenario no.* | | *RRs in  GS of interest* | *RRs in  complementary GS´* |  | *PDR Kendall^$^* | *DRP  Pearson* | *DOP Pearson* | *DSQP Pearson* | *PDR Spearman^$^* |
| H_0_: F(RR|GS)=F(RR|GS´) | | | | | | | | | |
| 1 | no gene is associated at all | 10x1 | 90x1 | 10 | 4.4% | 4.8% | 4.2% | 4.4% | 4.2% |
| 2 | all genes are associated | 10x1.2 | 90x1.2 | 10 | 7.0% | 7.2% | 5.6% | 5.0% | 6.0% |
| 3 | all genes are associated | 10x1.5 | 90x1.5 | 10 | 4.8% | 5.4% | 4.0% | 4.2% | 5.0% |
| H_A_: RR>1 only in GS / F(RR|GS)≠F(RR|GS´) | | | | | | | | | |
| 4 | ½ the genes in GS are associated | 5x1 5x1.1 | 90x1 | 10 | 9.2% | 5.0% | 9.2% | 7.6% | 7.8% |
| 5 | | 5x1 5x1.2 | 90x1 | 10 | 26.4% | 26.0% | 16.4% | 21.8% | 26.6% |
| 6 | | 5x1 5x1.3 | 90x1 | 10 | 48.8% | 50.8% | 22.0% | 35.0% | 50.0% |
| 7 | | 5x1 5x1.4 | 90x1 | 10 | 58.0% | 55.6% | 17.4% | 33.0% | 59.0% |
| 8 | | 5x1 5x1.5 | 90x1 | 10 | 58.8% | 57.6% | 16.0% | 32.0% | 59.4% |
| H_A_: RR>1 only in GS / F(RR|S)≠F(RR|NS) – increasing number of studies | | | | | | | | | |
| 9 | | 5x1 5x1.5 | 90x1 | 2 | 24.4% | 24.4% | 14.6% | 17.8% | 26.2% |
| 10 | | 5x1 5x1.5 | 90x1 | 3 | 31.2% | 30.0% | 20.4% | 23.6% | 33.6% |
| 11 | | 5x1 5x1.5 | 90x1 | 4 | 41.8% | 41.2% | 16.8% | 24.8% | 44.0% |
| 12 | | 5x1 5x1.5 | 90x1 | 5 | 45.0% | 43.6% | 19.4% | 28.2% | 46.0% |
| 13 | | 5x1 5x1.5 | 90x1 | 6 | 47.4% | 48.2% | 16.2% | 28.8% | 48.4% |
| 14 | | 5x1 5x1.5 | 90x1 | 7 | 50.4% | 50.6% | 15.6% | 29.2% | 52.2% |
| 15 | | 5x1 5x1.5 | 90x1 | 8 | 54.4% | 53.4% | 17.6% | 29.6% | 54.8% |
| 16 | | 5x1 5x1.5 | 90x1 | 9 | 55.2% | 54.2% | 17.2% | 29.6% | 56.6% |
| H_A_/H_0_: mixed structured GS | | | | | | | | | |
| 17 | H_A_: assoc. genes in GS only | 5x1 4x1.2 1x1.5 | 90x1 | 10 | 35.6% | 33.8% | 20.6% | 25.6% | 35.8% |
| 18 | H_A_: GS dominats GS’ | 5x1 4x1.2 1x1.5 | 63x1 18x1.2 9x1.5 | 10 | 5.8% | 6.0% | 5.6% | 4.4% | 5.6% |
| 19 | H_A_: GS is dominated**^§^** by GS‘ | 5x1 4x1.2 1x1.5 | 41x1 32x1.2 17x1.5 | 10 | 1.6% | 1.4% | 3.0% | 2.4% | 1.4% |
| 20 | H_0_: same prop. of genes are associated in GS and GS’ | 5x1 4x1.2 1x1.5 | 45x1 36x1.2 9x1.5 | 10 | 3.0% | 2.6% | 4.8% | 2.6% | 2.8% |

^$^ The power is equal for all possible PDR equation (1,S1): DRP, (S2): DOP and (S3): DSQP; Given a true type I error of 5%, the observed type I error may range from 3% to 7% (95% random dispersion interval for 500 simulations). Given a true power of 50%, the observed power may range from 45% to 54% (95% random dispersion interval for 500 simulations). ^§^ Truly associated genes are more frequent in GS’ than in GS.
