# Supplementary material for: META-GSA: Combining Findings from Gene-Set Analyses across Several Genome-Wide Association Studies
Source: PLoS One. 2015 Oct 26;10(10):e0140179. doi: 10.1371/journal.pone.0140179 (PMC4621033; doi:10.1371/journal.pone.0140179)
Supplement: S3 Text — (DOCX) [file pone.0140179.s005.docx]

## Motivation to use r² as measure for LD between markers

Suppose that the representative association measure θ_I,1_ of study 1 derives from marker I with alleles A and a and frequencies f_A_ and f_a_ (the lower-case letter denotes the minor allele). Similarly, the representative association measure θ_II,2_ of study 2 derives from another marker II with alleles B and b and frequencies f_B_ and f_b_. Furthermore, suppose that the source population of both studies is the same and the haplotype frequencies h_AB_, h_Ab_, h_aB_ and h_ab_ are given.

The probability that both studies reveal associations in the same direction (both minor alleles tell us the same story) is proportional to $h_{AB}h_{ab}$. If the markers are in complete linkage disequilibrium (LD), this quantity takes on the value $f_{A}f_{B}f_{a}f_{b}$ [[1](#_ENREF_1)]. Hence, this quantity is sensitive to the observed marker allele frequencies.

A more robust measure than $h_{AB}h_{ab}$, which has almost the same values in the case of common minor alleles, is $r_{I,II}=\frac{h_{AB}h_{ab}-h_{Ab}h_{aB}}{\sqrt{f_{a}f_{b}(1-f_{a})(1-f_{b})}}$. This is simply the correlation coefficient for a 2×2 table [[2](#_ENREF_2)].

Reference

1. Pritchard JK, Przeworski M (2001) Linkage disequilibrium in humans: models and data. Am J Hum Genet 69: 1-14.

2. Devlin B, Risch N (1995) A comparison of linkage disequilibrium measures for fine-scale mapping. Genomics 29: 311-322.
