# Supplementary material for: META-GSA: Combining Findings from Gene-Set Analyses across Several Genome-Wide Association Studies
Source: PLoS One. 2015 Oct 26;10(10):e0140179. doi: 10.1371/journal.pone.0140179 (PMC4621033; doi:10.1371/journal.pone.0140179)
Supplement: S4 Text — (DOCX) [file pone.0140179.s006.docx]

## Performing significance testing

The statistical significance is expressed by the permutation p-value, which is assessed by the fraction of permutations achieving a test statistic $M_{j}$ (j=1 to x) at least as extreme as the non-permuted (original) test statistic M_0_. Given the *number of exceeding permutations* as $x^{*}=n\left( M_{j}\leq M_{0} \right)$, the exact p-values are fairly approximated by:

$p_{permut}={{(x}^{*}+1)}/{(x+1)}$ (S5)

Smyth and Phipson (2011) [[1](#_ENREF_1)] outlined that applying $p_{permut}={x^{*}}/x$ instead would lead to a small underestimation of the p-value, but this can get serious in the context of multiple testing. Furthermore, the possibility of $p_{permut}=0$ when $x^{*}=0$ is avoided using equation (8).

Reference

1. Phipson B, Smyth GK (2010) Permutation P-values should never be zero: calculating exact P-values when permutations are randomly drawn. Stat Appl Genet Mol Biol 9: Article39.
