# Supplementary material for: META-GSA: Combining Findings from Gene-Set Analyses across Several Genome-Wide Association Studies
Source: PLoS One. 2015 Oct 26;10(10):e0140179. doi: 10.1371/journal.pone.0140179 (PMC4621033; doi:10.1371/journal.pone.0140179)
Supplement: S5 Text — (DOCX) [file pone.0140179.s007.docx]

## Speeding up the permutation

Performing a permutation test can be time consuming and requires sufficient computing capacity, depending on the total number of genes in the GSA, the size of the gene sets, and the number of studies to be combined. To limit the computational burden, we took the following three steps:

1. We limited the number of permutation steps to a maximum of x_max_=10 000.
2. To increase the precision of achieved p-values of highly significant gene sets, the tail of the null distribution of M is approximated by a generalized Pareto distribution (GPD), as described by Knijnenburg [[1](#_ENREF_1)].
3. Early stopping of the permutation procedure is allowed if the chance of achieving a p-value around the level of significance α becomes low, as described by Gandy [[2](#_ENREF_2)]*.*

Performing a permutation test can be time consuming and requires sufficient computing capacity, depending on the total number of genes in the GSA, the size of the gene-sets and the number of studies to be combined. To limit the computational burden, some steps of efficient computing were taken:

### Limiting permutation steps

We limited the number of permutation steps to a maximum of x_max_=10 000. Thus, the exact 95% confidence interval of a p-value corresponding to the level of significance of α=0.05 (500 out of 10 000 times $M_{j}\leq M_{0}$) will be [0.0458, 0.0554]. Hence the p-values will be approximated with a fairly high precision of ~0.005, if assigning nominal significance can be critical.

### Distributional determination of extreme p-values

If the *number of exceeding permutations* $x^{*}$ is low ($x^{*}\leq50$ out of 10 000 permutations), the precision of estimated p-values, which will indicate highly significant gene-sets, is low too. Instead of increasing the number of permutations to achieve more reliable p-values in such a case, the tail of the null distribution of M is therefore approximated by a Generalized Pareto Distribution (GPD), as described by Knijnenburg [[1](#_ENREF_1)]. In such a way accurate p-value estimates can be obtained with a drastically reduced number of permutations.

Let denote *x_used_* the number of the largest permuted test statistic $M_{j}$. The permutation test p-value is thereupon computed as $p_{permut}={x_{used}}/x\cdot\left( 1-Q_{x^{*}} \right)$, where *Q_x*_* is the *x**-quantile and the fraction *x_used_/x* compensates for the fact that *Q_x*_* is estimated only on the tail of the distribution of permutation values.

Because in the tail of the distribution one is dealing with the most significant gene-sets, care needs to be taken regarding the robustness of the approximation. Thus, deviating from the recommended *x_used_*=250 [[1](#_ENREF_1)], we searched for the best fitting GDP by changing *x_used_* between 100 and 500 in steps of 25. Goodness of fit tests (GOF) were performed to choose the most appropriate *x_used_*. If fitting a GPD failed, a corresponding exponential distribution (special case of GPD) was fitted instead.

### Adequate stopping rule

Although having set an absolute maximum of the number of permutation steps, we further limited the number of necessary permutation steps according to the *algorithm of sequential Monte Carlo testing [*[*2*](#_ENREF_2)*]*, which controls the *resampling risk* ${RR}_{p}$ to achieve a significant result by continuing the permutation procedure, defined as:

${RR}_{p}\left( \hat{p}_{permut} \right)= \left\{ \begin{matrix} p_{permut}\left( p<\alpha\right) & if p\leq\alpha\\ p_{permut}\left( p\leq\alpha\right) & if p>\alpha\end{matrix} \right.,$

where *p* indicates the p-value that could be achieved by infinite permuting.

Early stopping should be allowed if the chance to achieve a p-value around the level of significance α becomes low. The algorithm defines a region $\left[ L^{'},U^{'} \right]$of the smallest resampling risk${sup}_{p \in\left[ 0,1 \right]}{RR}_{p}\leq\epsilon_{x^{'}}$for each permutation step$x^{'}\leq x$. Multiple inspection is accounted for by applying the recommended spending function$\varepsilon_{x^{'}}=\varepsilon\frac{x}{1000+x^{'}}$ setting ε=0.01. Thus the permutation procedure will be stopped early for clearly insignificant and for highly significant pathways (see Supplementary Figure 4). Because local and multiple corrected *p_permut_*–values for gene-sets are of interest, we determined the upper boundary for stopping (*U’*) for α=0.05 and a lower boundary (*L’*) for α=0.05/*n_GS_* (Bonferroni corrected).

Supplementary Figure 4: Stopping bounds U (for α=0.05) and L (for α=0.05/50).

permutation continued

permutation stopped

permutation stopped

Reference

1. Knijnenburg TA, Wessels LFA, Reinders MJT, Shmulevich I (2009) Fewer permutations, more accurate P-values. Bioinformatics 25: i161-i168.

2. Gandy A (2009) Sequential Implementation of Monte Carlo Tests With Uniformly Bounded Resampling Risk. Journal of the American Statistical Association 104: 1504-1511.
