# Supplementary material for: META-GSA: Combining Findings from Gene-Set Analyses across Several Genome-Wide Association Studies
Source: PLoS One. 2015 Oct 26;10(10):e0140179. doi: 10.1371/journal.pone.0140179 (PMC4621033; doi:10.1371/journal.pone.0140179)
Supplement: S6 Text — (DOCX) [file pone.0140179.s008.docx]

## Correction for multiple testing (the bootstrap method of Storey and Tibshirani)

We propose applying the bootstrap method of Storey and Tibshirani [[1](#_ENREF_1),[2](#_ENREF_2)] to estimate the proportion of true null hypotheses $\pi_{0}$ and use this in a Bonferroni-like manner to correct $p_{permute,GS}$ for multiple testing.

The method of Storey and Tibshirani makes use of the vector of resulting *p_permut,GS_*-values to determine a tuning parameter λ, in order to estimate the proportion of true null hypotheses $\pi_{0}(\lambda)=\frac{n_{GS}-n_{GS}^{*}\left( \lambda\right)+z}{\left( 1-\lambda\right)n_{GS}}$ for $\lambda\in L= \left\{ 0,\frac{1}{n_{GS}},\ldots,\frac{n_{GS}-1}{n_{GS}} \right\}$, where $n_{GS}^{*}$ is the number of *p_permut,GS_*-values less than or equal to λ and z*=1* for the finite-sample case; otherwise z*=0*. For each λ, we bootstrap on the p-values to form *B* bootstrap versions $\hat{\pi}_{0}^{b}\left( \lambda\right)$, with *b=1,…, B*, and choose the “optimum” λ that yields the minimum $\hat{MSE}\left( \lambda\right)=\frac{1}{B}\sum_{b=1}^{B} \left( \hat{\pi}_{0}^{b}\left( \lambda\right)-{min}_{\lambda^{'}\in L}\hat{\pi}_{0}(\lambda^{'}) \right)^{2}$.

Finally the corrected p-value for each GS is calculated as:

$p_{META-GSA,GS}=min(1,p_{permute,GS}\cdot n_{GS}\cdot\pi_{0}\left( \lambda\right))$ (S6)

### Correction for multiple: Application: GO-pathways and lung cancer

Of the 421 investigated gene sets, 313 belong to *biological processes,* according to the GO hierarchy, while the remaining 108 gene sets belong to *molecular functions*. To maintain the balance between these two functional classes, we used a weighted Bonferroni adjustment to correct for multiple testing [[3](#_ENREF_3)]. In general, a null-hypothesis H_0i_ of no association of gene set *i* (*i=1 to n_GS_*) is rejected if: $\frac{p_{i}}{w_{i}}\leq\frac{\alpha}{t}$ as long as $\sum_{i=1}^{n} w_{i}=t$, where *α*=0.05 is the global level of significance, *t* is the effective number of tests, and *w_i_* is a weight assigned to each test *i* [[3](#_ENREF_3)]. Reformulated, the p-values of META-GSA adjusted for multiple testing are given as $p_{META-GSA,GS}=\min\left( 1,{\frac{t}{w_{GS}}p}_{permut,GS} \right)$. We defined equal weights for each gene set within a function class as $w_{GS,f}=\frac{t}{2t_{f}}$, with *t_f_* the effective number of gene sets of the function class and $t=\sum_{f} t_{f}$.

References

1. Storey JD, Tibshirani R (2003) Statistical significance for genomewide studies. Proc Natl Acad Sci USA 100: 9440-9445.

2. Publishing SAS (2010) SAS/Stat 9.22 User's Guide the MULTTEST Procedure: SAS Publishing.

3. Kang G, Ye K, Liu N, Allison DB, Gao G (2009) Weighted multiple hypothesis testing procedures. Stat Appl Genet Mol Biol 8: Article23.
