# Supplementary material for: META-GSA: Combining Findings from Gene-Set Analyses across Several Genome-Wide Association Studies
Source: PLoS One. 2015 Oct 26;10(10):e0140179. doi: 10.1371/journal.pone.0140179 (PMC4621033; doi:10.1371/journal.pone.0140179)
Supplement: S7 Text — (DOCX) [file pone.0140179.s009.docx]

## Comparison META-GSA and SPP in the application

Supplementary Figure 5: Comparison of p-values by GSA-method


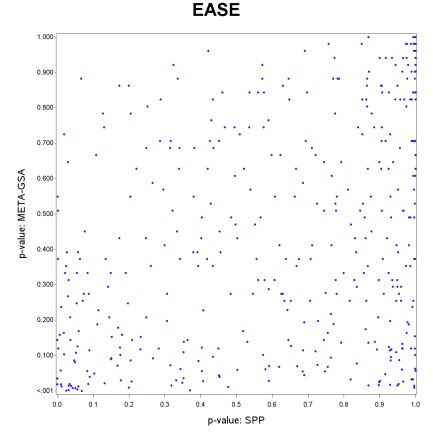

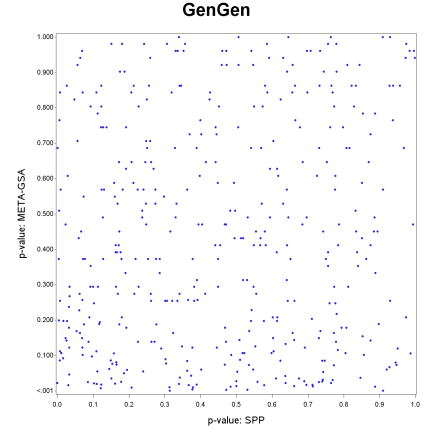

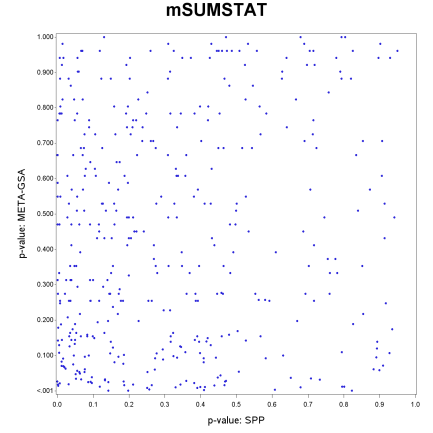

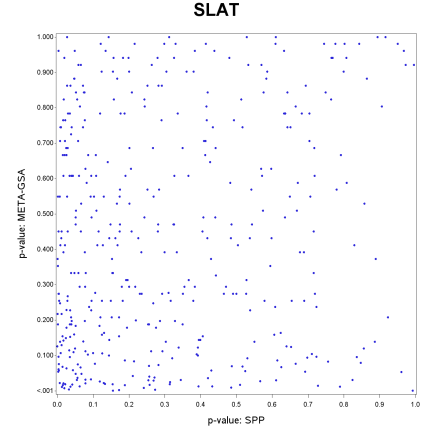


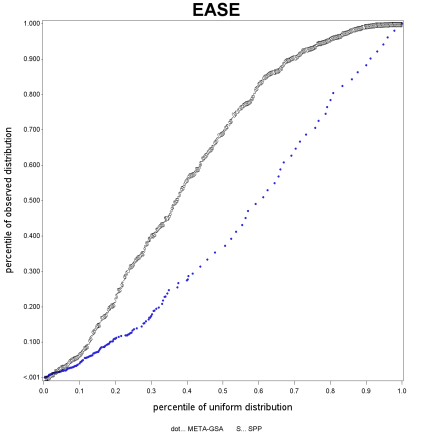

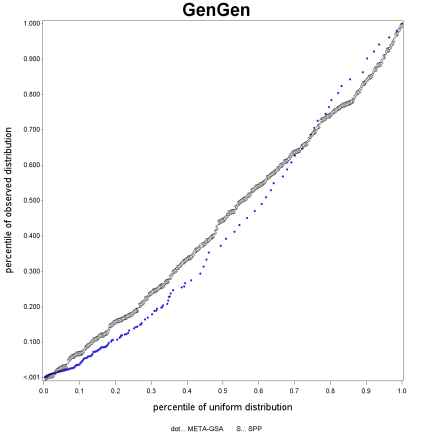

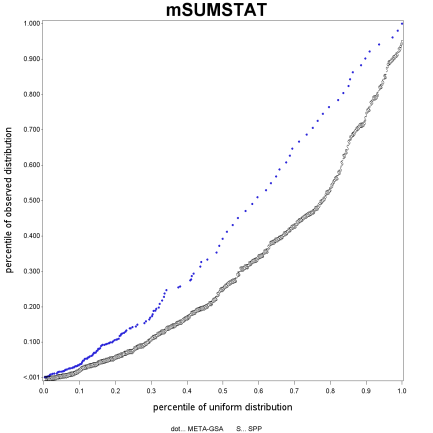

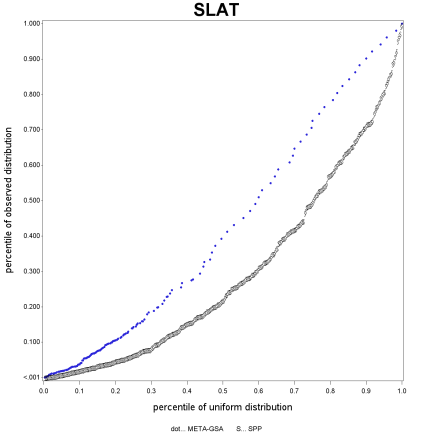


ρ=0.144

ρ=0.109

ρ=0.098

ρ=0.358

*upper row*: direct comparison of p-values (META-GSA vs. SPP) – Spearman’s correlation coefficient ρ is give ;
*lower row*: comparative QQ-plots (assumed uniform distribution); all markers of a gene were used to determine concordance.

The plots of the upper row illustrate the low correlation of p-values from META-GSA and SPP, respectively. This can be expected; since no accumulated association can be assumed for most of the investigated gene-sets (these plots are similar to Fig. 2). However, a weak clustering of low p-values (for META-GSA and SPP) can see for EASE, mSUMSTAT and SLAT. The QQ-plots in the lower row illustrate the harmonizing effect of META-GSA (dotted lines) and the strong heterogeneity in results across GSA-methods.
