# Supplementary material for: META-GSA: Combining Findings from Gene-Set Analyses across Several Genome-Wide Association Studies
Source: PLoS One. 2015 Oct 26;10(10):e0140179. doi: 10.1371/journal.pone.0140179 (PMC4621033; doi:10.1371/journal.pone.0140179)
Supplement: S8 Text — (DOCX) [file pone.0140179.s010.docx]

## A “quick but dirty” test

We also developed a quick but dirty pre-test (QbdT) in order to weed out pathways with only a small chance of turning out significant. For this test *significance* and *concordance* are marginal combined instead of weighting *significance* by *concordance* for each study

For *significance* of the GS, we now use Fisher’s inverse χ²-method with *df=2n_s_* to combine single p-values (*p_GS,s_*), which we previously discussed as inadequate, yielding *p_SPP_*.

For *concordance* we first calculate the mean of directions of each gene $\bar{d}_{g}$ across studies. The absolute value $abs\left( d_{g,s} \right)$ will only be large if all directions are close to either +1 or -1. This $abs\left( d_{g,s} \right)$ will tend to be small if the directions are close to 0 or inhomogeneous. Thus, we perform a one-sided Wilcoxon rank-sum test of $abs\left( d_{g,s} \right)$-values, contrasting genes of GS and GS´, yielding *p_direction_*.

Finally, we apply Fisher’s inverse χ²-method again with *df=4* to combine *p_SPP_* and *p_direction_*: $M_{QbdT}=-2\left[ ln(p_{SSP})+ln(p_{direction}) \right]$ and $p_{QbdT}=p(M_{QbdT}\geq\chi_{df=4}^{2})$:

This Qbd-test should not regarded as a valid alternative test to META-GSA because the two independent elements, the simple combination of *p_GSA,s_* into *p_SPP_* and the marginal combination of evidence for significance and concordance instead of weighting *significance* by *concordance* for each study, are inadequate. Avoiding unnecessary permutations is the sole purpose of the Qbd-test.

### META-GSA vs. QbdT

Comparing META-GSA with other considered tests reveals the strongest correlations of *p_META-GSA_*-values with *p_direction_*-values (ρ > 0.75) and less with *p_QbdT_*-values (ρ ≈ 0.5) and *p_SPP_*-values (ρ ≈ 0.1). In the considered data, *p_direction_*-values and $p_{SPP}$-values were almost completely uncorrelated (ρ ≈ 0.1, see Supplementary Table 2).

Supplementary Table 2: Rank correlation of p-values comparing META-GSA, SPP, direction test and Qbd-test by GSA methods (best SNP approach)

|  | *GenGen* | *EASE* | *SLAT* | *mSUMSTAT* |
| --- | --- | --- | --- | --- |
| $\boldsymbol{p}_{\boldsymbol{META\_GSA}}$ vs. $\boldsymbol{p}_{\boldsymbol{direction}}$ | 0.80* | 0.75* | 0.79* | 0.78* |
| $\boldsymbol{p}_{\boldsymbol{META\_GSA}}$ vs. $\boldsymbol{p}_{\boldsymbol{Qbdt}}$ | 0.68* | 0.59* | 0.61* | 0.58* |
| $\boldsymbol{p}_{\boldsymbol{META\_GSA}}$ vs. $\boldsymbol{p}_{\boldsymbol{SPP}}$ | 0.12* | 0.05 | 0.13* | 0.10* |
| $\boldsymbol{p}_{\boldsymbol{SPP}}$ vs. $\boldsymbol{p}_{\boldsymbol{direction}}$ | 0.09 | 0.08 | 0.11* | 0.11* |

* significantly different from ρ=0

### Validity of the Qbd-test

To assess the validity of the *quick but dirty test*, we contrasted permutation p-values of the META-GSA with p-values of the Qbd-test. For GenGen-results, when only the most significant marker at a gene was used, 392 out of the 421 investigated pathways yielded a non-significant *p_META‑GSA_*> 0.05. According to the Qbd-test, 354 of these pathways could have been correctly weeded out when employing a threshold of 0.05. This is a specificity of approximately 90%. Twenty-nine pathways were nominally significant (*p_META-GSA_*≤0.05). According to the Qbd-test, only 4 of these pathways would have been falsely weeded out, which can be expressed as a sensitivity of about 86%. All in all, only 63 of 421 (or one out of six) pathways would have been included in the time-consuming permutation test (see Supplementary Table 1)).

To increase the sensitivity up to 99% (and therefore almost avoid false exclusions), a threshold of *p_QbdT_* = 0.74 is needed. However, the specificity would drop to 12% at the same time and 372 pathways (about eight out of nine) would enter the permutation procedure. Nevertheless, the reduction in computational burden is limited even for those gene sets which would be sorted out, because most of these would already have been found as not significant in the first 50 or 100 permutation circles by the applied stopping rule procedure (see Supplementary Table 1).

Supplementary Table 3: Validity of the Qbd-test assessed by results of META-GSA as gold standard

|  | *GenGen (n=421)* | | | *EASE (n=421)* | | | *SLAT (n=420)* | | | *mSUMSTAT (n=421)* | | |
| --- | --- | --- | --- | --- | --- | --- | --- | --- | --- | --- | --- | --- |
| *Inclusion for permutation procedure based on a cutoff of* $\boldsymbol{p}_{\boldsymbol{Qbdt}}\boldsymbol{=0.05}$ | | | | | | | | | | | | |
|  | $\boldsymbol{p}_{\boldsymbol{META-GSA}}$ $\boldsymbol{\leq}0.05$ | $\boldsymbol{p}_{\boldsymbol{META-GSA}}$ $\boldsymbol{>}0.05$ | | $\boldsymbol{p}_{\boldsymbol{META-GSA}}$ $\boldsymbol{\leq}0.05$ | $\boldsymbol{p}_{\boldsymbol{META-GSA}}$ $\boldsymbol{>}0.05$ | | $\boldsymbol{p}_{\boldsymbol{META-GSA}}$ $\boldsymbol{\leq}0.05$ | $\boldsymbol{p}_{\boldsymbol{META-GSA}}$ $\boldsymbol{>}0.05$ | | $\boldsymbol{p}_{\boldsymbol{META-GSA}}$ $\boldsymbol{\leq}0.05$ | $\boldsymbol{p}_{\boldsymbol{META-GSA}}$ $\boldsymbol{>}0.05$ | |
| *further included* $\boldsymbol{p}_{\boldsymbol{Qbdt}}\boldsymbol{\leq0.05}$ | 25 86% | 38 | | 23 85% | 35 | | 26 93% | 85 | | 27 90% | 78 | |
| *excluded* $\boldsymbol{p}_{\boldsymbol{Qbdt}}\boldsymbol{>0.05}$ | 4 | 354 90% | | 4 | 359 91% | | 2 | 307 78% | | 3 | 313 80% | |
| *total* | ***29*** | ***392*** | | ***27*** | ***394*** | | ***28*** | ***392*** | | ***30*** | ***391*** | |
| *cut-offs (*$\boldsymbol{p}_{\boldsymbol{Qbdt}}$*) for Qbd-test to achieve a sensitivity of …* | | | | | | | | | | | | |
| *sensitivity* | $\boldsymbol{p}_{\boldsymbol{Qbdt}}$ | ***spec*** | ***n_excluded_*** | $\boldsymbol{p}_{\boldsymbol{Qbdt}}$ | ***spec*** | ***n_excluded_*** | $\boldsymbol{p}_{\boldsymbol{Qbdt}}$ | ***spec*** | ***n_excluded_*** | $\boldsymbol{p}_{\boldsymbol{Qbdt}}$ | ***spec*** | ***n_excluded_*** |
| 90% | 0.06544 | 88% | 343 | 0.05729 | 85% | 337 | 0.01230 | 89% | 350 | 0.04474 | 78% | 305 |
| 95% | 0.08159 | 62% | 244 | 0.16673 | 50% | 195 | 0.04121 | 76% | 297 | 0.46381 | 22% | 85 |
| 99% | 0.73669 | 12% | 49 | 0.78881 | 9% | 37 | 0.10494 | 59% | 232 | 0.93897 | 1% | 5 |

*specificity (spec, true exclusion rate), expected number of excluded gene sets (n_excluded_)*
