# Supplementary material for: META-GSA: Combining Findings from Gene-Set Analyses across Several Genome-Wide Association Studies
Source: PLoS One. 2015 Oct 26;10(10):e0140179. doi: 10.1371/journal.pone.0140179 (PMC4621033; doi:10.1371/journal.pone.0140179)
Supplement: S9 Text — (DOCX) [file pone.0140179.s011.docx]

| **Section/topic** | **#** | **Checklist item** | **Reported on page #** |
| --- | --- | --- | --- |
| **TITLE** | | |  |
| Title | 1 | Meta-GSA: Combining findings from gene-set analyses across several genome-wide association studies. |  |
| **ABSTRACT** | | |  |
| Structured summary | 2 | Gene-set analysis (GSA) methods are used as complementary approaches to genome-wide association studies (GWASs). The single marker association estimates of a predefined set of genes are either contrasted with those of all remaining genes or with a null non-associated background. To pool the p-values from several GSAs, it is important to take into account the concordance of the observed patterns resulting from single marker association point estimates across any given gene set. Here we propose an enhanced version of Fisher’s inverse χ²-method META-GSA, however weighting each study to account for imperfect correlation between association patterns.  We investigated the performance of META-GSA by simulating GWASs with 500 cases and 500 controls at 100 diallelic markers in 20 different scenarios, simulating different relative risks between 1 and 1.5 in gene sets of 10 genes. Wilcoxon’s rank sum test was applied as GSA for each study. We found that META-GSA has greater power to discover truly associated gene sets than simple pooling of the p-values, by e.g. 59% versus 37%, when the true relative risk for 5 of 10 genes was assume to be 1.5. Under the null hypothesis of no difference in the true association pattern between the gene set of interest and the set of remaining genes, the results of both approaches are almost uncorrelated. We recommend not relying on p-values alone when combining the results of independent GSAs.  We applied META-GSA to pool the results of four case-control GWASs of lung cancer risk (Central European Study and Toronto/Samuel Lunenfeld Research Institute Study; German Lung Cancer Study and MD Anderson Cancer Center Study), which had already been analyzed separately with four different GSA methods (EASE; SLAT, mSUMSTAT and GenGen). This application revealed the pathway GO0015291 “transmembrane transporter activity” as significantly enriched with associated genes (GSA-method: EASE, p=0.0315 corrected for multiple testing). Similar results were found for GO0015464 “acetylcholine receptor activity” but only when not corrected for multiple testing (all GSA-methods applied; p≈0.02). | 2 |
| **INTRODUCTION** | | |  |
| Rationale | 3 | This is the first and only exemplary meta-analysis of |  |
| Objectives | 4 | **Questions being addressed**: Identification of predefined sets of gens, being enriched across studies with genes associated to lung cancer at a nominal significance level, accounting for correlation across studies in the patterns resulting from single marker association point estimates  **Participants**: Lung cancer patients and hospital or population controls  **Intervention**: non  **Comparisons**: at the GSA level: “gene set of interest” with all “remaining genes” at the GWAS level: cases with controls  **Outcomes**: p-values  **Study design:** Meta-analysis of gene-set analyses (GSA) based on genome-wide association studies (GWASs). | 19-20 |
| **METHODS** | | |  |
| Protocol and registration | 5 | no review protocol exists, because a reassignment of significance of a previously reported combination of four studies was presented | 19 |
| Eligibility criteria | 6 | Already performed GSA based on GWAS addressing lung cancer with at least 300 cases and 300 controls | 19 |
| Information sources | 7 | Data shared within the *International Lung Cancer Consortium (ILCCO)* | 19 |
| Search | 8 | -- |  |
| Study selection | 9 | -- |  |
| Data collection process | 10 | All studies had previously been used to compare the performance of four different GSA approaches by Fehringer Get al. (2012) PLoS One 7: e31816 | 19 |
| Data items | 11 | GWAS: odds ratio and p-value for single marker association test , snp-to-gene allocation;  GSA, p-values and gene-to-pathway allocation |  |
| Risk of bias in individual studies | 12 | GWAS: any remaining risk of case-control studies, after careful accomplishment GSA: insufficient marker coverage and insufficient calculation of gene-level statistics |  |
| Summary measures | 13 | p-value |  |
| Synthesis of results | 14 | described in detail in the manuscript | 21-23 |

Page 1 of 2

| **Section/topic** | **#** | **Checklist item** | **Reported on page #** |
| --- | --- | --- | --- |
| Risk of bias across studies | 15 | restricted study selection one of the four studies investigated small cell lung cancer patients only another of the four studies focused on young cases and controls (<50 years of age) |  |
| Additional analyses | 16 | -- |  |
| **RESULTS** | | |  |
| Study selection | 17 | -- |  |
| Study characteristics | 18 | Study characteristics are given in detail in: Fehringer G, Liu G, Briollais L, Brennan P, Amos CI, et al. (2012) Comparison of Pathway Analysis Approaches Using Lung Cancer GWAS Data Sets. PLoS One 7: e31816 | 20 |
| Risk of bias within studies | 19 | -- |  |
| Results of individual studies | 20 | -- |  |
| Synthesis of results | 21 | GO0015291 “transmembrane transporter activity” (GSA-method: EASE): *p*=0.0315 GO0015464 “acetylcholine receptor activity” (all GSA-methods applied): *p*≈0.02 (not adjusted for multiple testing) | 22,34 |
| Risk of bias across studies | 22 | -- |  |
| Additional analysis | 23 | -- |  |
| **DISCUSSION** | | |  |
| Summary of evidence | 24 | -- |  |
| Limitations | 25 | restricted study selection |  |
| Conclusions | 26 | -- |  |
| **FUNDING** | | |  |
| Funding | 27 | The investigations in this article were supported by the grant from the National Institute of Health (NIH) (U19CA148127). The funding has no impact onto the results. |  |

Page 2 of 2
