# Supplementary material for: META-GSA: Combining Findings from Gene-Set Analyses across Several Genome-Wide Association Studies
Source: PLoS One. 2015 Oct 26;10(10):e0140179. doi: 10.1371/journal.pone.0140179 (PMC4621033; doi:10.1371/journal.pone.0140179)
Supplement: S10 Text — (DOCX) [file pone.0140179.s012.docx]

Studies included in quantitative synthesis (meta-analysis)
(n = 4 )

Studies included in qualitative synthesis
(n = 4 )

Full-text articles excluded, with reasons
(n = 0 )

Full-text articles assessed for eligibility
(n = 0 )

Records excluded
(n =0 )

Records screened
(n = 4 )

Records after duplicates removed
(n = 0 )

Additional records identified through other sources
(n = 4 )

## Identification

## Eligibility

## Included

## Screening

Records identified through database searching
(n = 0 )
